# Conservative and disruptive modes of adolescent change in human brain functional connectivity

**DOI:** 10.1073/pnas.1906144117

**Published:** 2020-01-28

**Authors:** František Váša, Rafael Romero-Garcia, Manfred G. Kitzbichler, Jakob Seidlitz, Kirstie J. Whitaker, Matilde M. Vaghi, Prantik Kundu, Ameera X. Patel, Peter Fonagy, Raymond J. Dolan, Peter B. Jones, Ian M. Goodyer, the NSPN Consortium, Petra E. Vértes, Edward T. Bullmore

**Affiliations:** ^a^Department of Psychiatry, University of Cambridge, Cambridge CB2 0SZ, United Kingdom;; ^b^Department of Neuroimaging, Institute of Psychiatry, Psychology and Neurosciences, King’s College London, London SE5 8AF, United Kingdom;; ^c^Developmental Neurogenomics Unit, National Institute of Mental Health, Bethesda, MD 20892;; ^d^The Alan Turing Institute, London NW1 2DB, United Kingdom;; ^e^Wellcome Trust Centre for Neuroimaging, University College London Institute of Neurology, University College London, London WC1N 3BG, United Kingdom;; ^f^Max Planck University College London Centre for Computational Psychiatry and Ageing Research, University College London, London WC1B 5EH, United Kingdom;; ^g^Brain Imaging Center, Icahn School of Medicine at Mount Sinai, New York, NY 10029;; ^h^Research Department of Clinical, Educational and Health Psychology, University College London, London WC1E 6BT, United Kingdom;; ^i^Cambridgeshire and Peterborough National Health Service (NHS) Foundation Trust, Huntingdon PE29 3RJ, United Kingdom;; ^j^School of Mathematical Sciences, Queen Mary University of London, London E1 4NS, United Kingdom

**Keywords:** neurodevelopment, connectome, MRI, Allen Human Brain Atlas, head movement

## Abstract

Adolescent changes in human brain function are not entirely understood. Here, we used multiecho functional MRI (fMRI) to measure developmental change in functional connectivity (FC) of resting-state oscillations between pairs of 330 cortical regions and 16 subcortical regions in 298 healthy adolescents scanned 520 times. Participants were aged 14 to 26 y and were scanned on 1 to 3 occasions at least 6 mo apart. We found 2 distinct modes of age-related change in FC: “conservative” and “disruptive.” Conservative development was characteristic of primary cortex, which was strongly connected at 14 y and became even more connected in the period from 14 to 26 y. Disruptive development was characteristic of association cortex and subcortical regions, where connectivity was remodeled: connections that were weak at 14 y became stronger during adolescence, and connections that were strong at 14 y became weaker. These modes of development were quantified using the maturational index (MI), estimated as Spearman’s correlation between edgewise baseline FC (at 14 y, $FC_{14}$) and adolescent change in FC ($\Delta FC_{14-26}$), at each region. Disruptive systems (with negative MI) were activated by social cognition and autobiographical memory tasks in prior fMRI data and significantly colocated with prior maps of aerobic glycolysis (AG), AG-related gene expression, postnatal cortical surface expansion, and adolescent shrinkage of cortical thickness. The presence of these 2 modes of development was robust to numerous sensitivity analyses. We conclude that human brain organization is disrupted during adolescence by remodeling of FC between association cortical and subcortical areas.

## Significance

How does the human brain change during adolescence? We found 2 distinct modes of change in functional connectivity between brain regions, “conservative” and “disruptive,” measured using functional MRI (fMRI) in healthy young people (14 to 26 y old). Conservative regions, often specialized for basic sensory and motor functions, were strongly connected at age 14 before strengthening more by age 26, whereas disruptive regions that were activated by complex tasks comprised both connections that were weak at age 14 but strengthened by age 26 and connections that were strong at age 14 but weakened by age 26. Disruptive maturation of fMRI connectivity between cortex and subcortex could represent metabolically costly remodeling that underpins development of adult faculties.

During adolescence, the human brain undergoes substantial changes in both structure (1, 2) and function (3, 4). Accurately describing these maturational processes is key to understanding the parallel changes in cognition and behavior as well as the vulnerability to mental health disorders (5) that characterize this critical developmental period.

Functional brain networks derived from functional MRI (fMRI) have proven to be useful for understanding large-scale brain organization (6, 7). The nodes of these fMRI networks correspond to macroscopic brain regions, and the edges correspond to the correlations in brain activity, or so-called functional connectivity (FC), between pairs of regionally localized, low-frequency oscillations. Several studies have reported age-related changes in functional brain networks during adolescence, but the findings are overall somewhat inconsistent. This is likely due in part to small sample sizes, the lack of longitudinal data, and significant variation in fMRI data preprocessing and analysis methods (*SI Appendix*, Table S1). In addition, although subcortical nuclei are theoretically well-recognized components of frontal cortico-striato-thalamic circuits, subcortical connectivity has generally been measured only for a few nuclei or ignored altogether (*SI Appendix*, Table S2).

Multiple prior resting-state fMRI studies of human brain development in childhood and adolescence replicably reported an age-related increase in the strength of long-range connections accompanied by a decrease in the strength of short-range connections (8–11). Since long-range connections tend to be concentrated on association cortical areas involved in higher-order cognitive functions, these results were consistent with prior work suggesting that primary sensory and motor areas mature earlier in childhood, whereas association areas show relatively protracted maturation, extending into adolescence and early adulthood (1, 2, 12–14).

However, it has since become clear that these changes in FC attributed to age might have been confounded by the effects of in-scanner head motion (13, 15–17). It is now well recognized that small (<1 mm), transient head movements during scanning can bias estimation of correlations between fMRI time series, and this is a critical issue for developmental studies because younger participants may find it more difficult to remain stationary in the scanner.

Here, we measured resting-state FC maturation in an accelerated longitudinal study of 298 healthy adolescents, aged 14 to 26 y, scanned 1 to 3 times. To correct fMRI time series for effects of participant in-scanner motion, we used multiecho scans (18) denoised using multiecho independent component analysis (ME-ICA) (19, 20) to identify and discard components of fMRI time series unrelated to the blood-oxygen-level–dependent (BOLD) signal. We further corrected residual effects of head motion using linear regression and investigated robustness of our findings to head movement by extensive supplementary analyses. For each preprocessed fMRI dataset, we estimated the Pearson’s correlation between all pairs of regional mean time series from each of 330 cortical areas and 16 subcortical nuclei. We identified 2 modes of developmental change in fMRI connectivity defined by positive or negative maturational index (MI) and assessed the psychological and biological relevance of these so-called “conservative” or “disruptive” systems by metaanalysis of prior task-related fMRI data and by testing for anatomical colocation of the MI map with prior maps of cortical metabolism, gene expression, postnatal areal expansion, and adolescent cortical shrinkage.

## Results

### Head Movement.

A total of 36 scans were excluded by one or more quality control criteria, including high in-scanner motion [μ(FD) > 0.3 mm or *max*(FD) > 1.3 mm] (*SI Appendix*). Following scan exclusion, regional fMRI time series were available at 330 cortical areas and 16 subcortical structures for 298 participants (151 females) scanned a total of 520 times (*SI Appendix*, Fig. S1).

In these data, we found no evidence of an age-related change in head movement indexed by framewise displacement (FD) (15). However, there was a positive correlation between FC and head movement and also distance dependence of the correlation between FC and FD, which was greater when the distance between nodes was greater (*SI Appendix*, Fig. S2). These confounding effects of head movement on connectivity in ME-ICA preprocessed data were corrected by regressing FC on mean FD (21, 22). The residual (mean FD-corrected) estimates of FC were not significantly correlated with head motion, and there was no distance dependence of the relationship between residual FC and FD (*SI Appendix*, Fig. S2). We, therefore, used this movement correction pipeline of ME-ICA followed by FD regression as the basis for further analysis of FC. We subsequently confirmed that the results obtained from our main analysis (*n* = 520) were qualitatively and quantitatively consistent with the results obtained by a sensitivity analysis using only a subset of “low-motion” fMRI data (*n* = 182) that had been acquired without discernible head motion (FD < 0.2 mm for each of 100 consecutive volumes) (23) and analyzed without FD regression (*SI Appendix*, Figs. S24–S28, S36, and S37). To test robustness of our results to an alternative movement correction strategy, we also used global signal regression (GSR) for movement correction of the whole sample (*n* = 520) and obtained results that were qualitatively consistent and correlated with results obtained both from our main analysis and from the low-motion data (*SI Appendix*, Figs. S29–S37).

### Age-Related Change of Connectivity Strength.

**Fig. 1.**
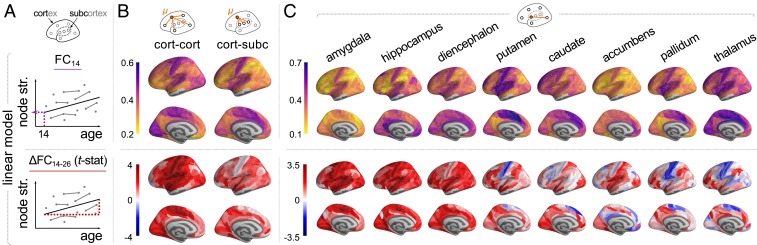
Regional strength of FC (weighted degree) of cortical areas and subcortical nuclei at 14 y ($FC_{14}$) and regional change in strength of connectivity during adolescence ($\Delta FC_{14-26}$). (*A*) Regional strength for each of 330 cortical and 16 subcortical nodes was regressed on a linear function of age for all participants (*n* = 520 scans from 298 participants; mixed effects model). (*B*) Parameters of cortico-cortical connectivity (*Left*) and cortico-subcortical connectivity (*Right*). Subcortico-cortical and subcortico-subcortical connectivity is in *SI Appendix*, Fig. S4. (*C*) Heterogeneous $FC_{14}$ and $\Delta FC_{14-26}$ of individual subcortical nuclei to cortex (subcortical regions are ordered by decreasing average rate of change). Due to bilateral symmetry and space constraints, only left hemispheres are visualized.

The FC, or weight of an edge between 2 nodes, as defined by the correlation between a pair of regional fMRI time series was generally positive. The global mean correlation weakly increased with age [*t*(221) = 2.3, *P* = 0.023] (*SI Appendix*, Fig. S3). For each regional node, we estimated its strength of connectivity (or weighted degree) by averaging the correlations between it and all other regions. We also calculated the strength of connectivity specifically within or between cortical and subcortical subsets of nodes. Using a mixed effect linear model of age-related change, we estimated the “baseline” strength of FC at age 14 y, $FC_{14}$, and the linear rate of change in weighted degree as a function of age, $\Delta FC_{14-26}$ (Fig. 1*A*), for each node. We also estimated the baseline and age-related change in FC for each edge.

At 14 y, all cortical regions had positive cortico-cortical connectivity strength, and the most strongly connected nodes were located in primary motor and sensory cortical areas. Cortico-subcortical connectivity strength had a similar anatomical distribution, with stronger connectivity between primary cortical areas and subcortex, at baseline (Fig. 1*B*). Age-related rates of change in connectivity strength were also regionally heterogeneous. Cortico-cortical connectivity strength increased in most regions during adolescence, most rapidly in primary motor and sensory cortex. However, age-related change in the strength of cortico-subcortical connectivity had a different anatomical distribution. The most positive rates of increase in connectivity were between subcortical nodes and association cortical areas, whereas some primary motor and sensory cortical areas had negative age-related changes in strength of connectivity with subcortical regions (Fig. 1*B*).

To further investigate cortico-subcortical connectivity, we estimated $FC_{14}$ and $\Delta FC_{14-26}$ between each cortical area and each bilateral pair of 8 subcortical regions (Fig. 1*C*). At baseline, the putamen, the pallidum, and the thalamus were strongly connected to many cortical areas, whereas the amygdala and the accumbens had somewhat lower strength of cortical connectivity overall. Over the course of adolescence, the amygdala (*P*_FDR_ < 0.05), the hippocampus (*P*_FDR_ < 0.05), and the diencephalon had increased cortical connectivity, whereas the putamen, the pallidum, and the thalamus had decreased strength of connectivity with primary somatomotor and premotor cortex but increased strength of connectivity to frontal and parietal association cortex. *SI Appendix*, Fig. S4 and Table S3 contain details.

### Maturational Index.

**Fig. 2.**
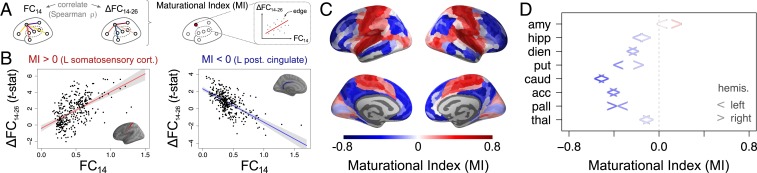
Maturational index (MI). (*A*) The MI for each brain region is defined as the correlation of edgewise baseline $FC_{14}$ vs. rate of change $\Delta FC_{14-26}$. (*B*) Estimation of MI is illustrated for 2 exemplar regions: left somatosensory cortex, which illustrates a “conservative" mode of development with positive MI, and left posterior cingulate cortex, which illustrates a “disruptive” mode of development with negative MI. (*C*) Visualization of the MI for all cortical regions and (*D*) subcortical regions (the left/right arrows correspond to the left/right hemispheres). acc, accumbens; amy, amygdala; caud, caudate; dien, diencephalon; hipp, hippocamus; pall, pallidum; put, putamen; thal, thalamus.

For each regional node, there was often a strong relationship between baseline connectivity $FC_{14}$ and adolescent change in connectivity $\Delta FC_{14-26}$ for the 345 edges connecting it to the rest of the network. We defined the MI as the signed coefficient (Spearman’s ρ) of the relationship between $FC_{14}$ and $\Delta FC_{14-26}$ for each node (Fig. 2*A*). MI was often significantly nonzero by statistical tests, including a permutation test controlling for regional contiguity and hemispheric symmetry (*P*_spin_) (*SI Appendix*, Fig. S5). For example, the left somatosensory cortex had strongly positive MI, indicating that the edges with strongest FC at baseline showed the greatest positive increase in FC during adolescence. Conversely, left posterior cingulate cortex had strongly negative MI, indicating that the edges with weakest FC at baseline showed the greatest positive increase in FC during adolescence (Fig. 2*B*). To put it another way, in somatosensory cortex and other regions with MI > 0, there was a conservative mode of developmental change: connections that were already strong at 14 become stronger by the age of 26, whereas in posterior cingulate cortex and other regions with MI < 0, there was a disruptive mode of developmental change: connections that were weak at 14 got stronger by the age of 26 (and connections that were strong at baseline became weaker) (Fig. 2 *C* and *D*).

**Fig. 3.**
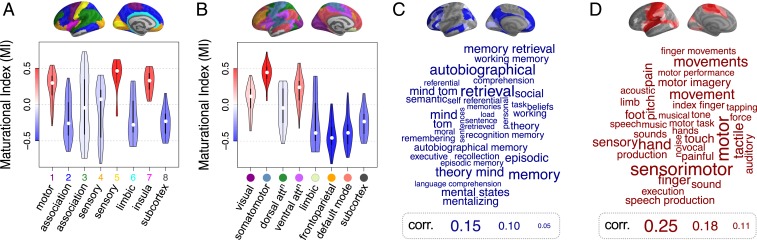
Maturational index (MI) in anatomical and psychological context. (*A*) Distribution of MI for each cytoarchitectonic class of the von Economo atlas (24) and (*B*) for resting-state networks derived from prior resting-state FC analysis by Yeo et al. (25). In both cases, subcortical regions were considered as an additional eighth class/subnetwork. The violin plots are colored by average MI within the corresponding class of regions. (*C* and *D*) Word clouds of cognitive terms associated with cortical brain regions that have (*C*) disruptive (blue) or (*D*) conservative (red) modes of development [Neurosynth decoding (26)]. The size of cognitive terms corresponds to the correlation of corresponding metaanalytic maps generated by Neurosynth with each of the 2 modes.

Conservative changes in connectivity were concentrated in primary motor and sensory areas corresponding to cytoarchitectonic classes 1 and 5 in the von Economo atlas (24) and the insula (Fig. 3*A*). This anatomical distribution maps onto motor, ventral attention, and visual networks previously defined by independent component analysis of adult resting-state fMRI data (Fig. 3*B*) (25). Disruptive changes in connectivity were concentrated in association cortex (von Economo class 2) and limbic cortex, corresponding to frontoparietal, default mode, and limbic resting-state networks. Subcortical nodes were almost all characterized by disruptive development, with weak baseline connectivity to association cortex becoming stronger and strong baseline connectivity to primary motor or sensory cortex becoming weaker (Fig. 2*D*).

To further characterize adolescent edgewise maturation of FC, we visualized average $FC_{14}$ and $FC_{14-26}$ within and between von Economo classes and functional networks, ascertained that $FC_{14-26}$ is not distance-dependent, and verified that most edgewise trajectories of FC are linear (*SI Appendix*, Figs. S6–S8).

### Contextualizing Adolescent Change in FC.

We used a metaanalytic tool (Neurosynth) (26) to identify cognitive processes or experimental task conditions that were associated with prior task-related activation of disruptively vs. conservatively developing cortical systems (Fig. 3 *C* and *D*). Disruptive changes in FC were located in cortical areas that were activated by memory, mentalizing, and social processing tasks. Conversely, conservative changes in FC were located in cortical areas that were activated by motor and sensory tasks.

**Fig. 4.**
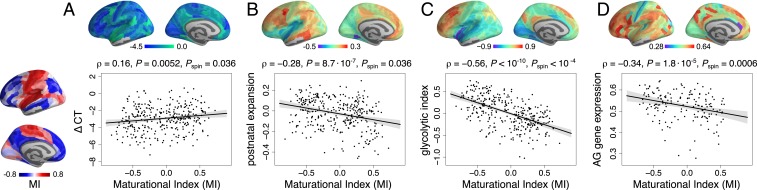
Disruptive and conservative modes of fMRI maturation in developmental and metabolic context. (*A*) MI was positively correlated with ΔCT (1)—regions that had disruptive development (MI < 0) had faster rates of cortical thickness (CT) shrinkage during adolescence. (*B*) MI was negatively correlated with a prior map of postnatal expansion of cortical surface area (28)—disruptive maturation was greater in regions that showed greatest expansion after birth. (*C*) MI was negatively correlated with a prior map of the glycolytic index, a measure of aerobic glycolysis (AG) (28), and (*D*) MI was negatively correlated with a prior map of brain regional expression of AG-related genes (29, 30).

We estimated cortical thickness shrinkage at each cortical node in a cross-sectional dataset of structural MRI scans collected from 297 of the participants in this fMRI study (1). The cortical areas with the most negative rates of thickness change (or fastest shrinkage) had the most negative MI (ρ = 0.16, *P* = 0.0052, *P*_spin_ = 0.036) (Fig. 4*A*). However, 2 other structural MRI markers of adolescent brain development were not significantly colocated with MI in this sample (*SI Appendix*, Fig. S9).

We further compared the MI map (Fig. 2*C*) with 9 independently produced maps of a range of brain functional and developmental parameters, including 1) evolutionary and postnatal surface expansion of the cortex (27); 2) metabolic rates of glucose, oxygen, and aerobic glycolysis (AG) measured by positron emission tomography (PET) (28); 3) microarray measures of gene expression for 116 genes previously associated with AG (14) and extracted from the Allen Human Brain Atlas (29) as in ref. 30; and 4) areal scaling of the cortical surface (31).

We found that disruptive cortical regions (with negative MI) had faster rates of postnatal surface expansion (ρ = −0.28, *P* = 8.7 ⋅ 10^−7^, *P*_spin_ = 0.036), higher metabolic rates of glucose (ρ = −0.41, *P* < 10^−10^, *P*_spin_ = 0.0032), higher rates of AG as measured by the glycolytic index (GI) (ρ = −0.56, *P* < 10^−10^, *P*_spin_ < 10^−4^), and higher expression of AG-related genes (ρ = −0.34, *P* = 1.8 ⋅ 10^−5^, *P*_spin_ = 0.0006) (Fig. 4 *B*–*D*).

All *P* values reported above were corrected for a total of 12 multiple comparisons using the false discovery rate. Details are in Fig. 4 and *SI Appendix*, Fig. S9 and Table S4.

### Sensitivity Analyses.

To evaluate the robustness of our results, we verified that the MI is consistent when edgewise $FC_{14}$ and $\Delta FC_{14-26}$ are derived from 1,000 sets of independent random half-splits of the data (2 × 260 scans) and when MI components are separately derived using cortico-cortical and subcortico-subcortical edges only (to account for potential differences between cortical and subcortical temporal signal-to-noise ratio [tSNR]) (*SI Appendix*, Figs. S10 and S11).

Further, we repeated main analyses (Figs. 1–4) under 5 conditions: 1) using a different cortical parcellation (*SI Appendix*, Figs. S12–S15); 2) in a subset of 298 cross-sectional scans (to rule out longitudinal effects of “regression to the mean”) (*SI Appendix*, Figs. S16–S19); 3) in a subset of 396 scans from a single scanner (to rule out scanner site effects) (*SI Appendix*, Figs. S20–S23); 4) in a subset of low-motion time series from 182 scans, displaying no discernible motion (*SI Appendix*, Figs. S24–S28); and 5) in the whole sample preprocessed using GSR (*SI Appendix*, Figs. S29–S35). In all cases, the following key results of the main analysis were recapitulated: 1) 2 modes of adolescent change in FC were defined by positive and negative MI; 2) conservatively maturing brain systems, defined by MI > 0, were concentrated in primary cortical areas, and disruptively maturing brain systems, defined by MI < 0, were concentrated in subcortical and association cortical areas; 3) disruptively maturing systems were significantly colocated with prior maps of AG and AG-related gene expression. Additionally, $FC_{14}$, $\Delta FC_{14-26}$ and MI metrics were positively correlated between the main analysis and the sensitivity analyses of GSR preprocessed data and a low-motion subset of data (*SI Appendix*, Figs. S36 and S37).

## Discussion

We have reported results from an accelerated longitudinal study of adolescent development of FC in the healthy human brain. In a large, population-representative sample of resting-state fMRI data balanced for age and sex and controlled for head motion, we found evidence for 2 modes of maturational change in the age range 14 to 26 y, which we called conservative and disruptive.

The conservative mode of change was consolidating, or making stronger over the course of adolescence, the connectivity of specialized sensory or motor cortical areas that were already highly connected at age 14. Conservatively, “the rich get richer.” In contrast, the disruptive mode of change was to make connectivity stronger in areas where it was relatively weak at age 14 or to make it weaker where it was relatively strong at the start of adolescence. Disruptively, “the rich get poorer, and the poor get richer.” Disruptive maturation was characteristic of association and limbic cortex, corresponding to default mode, frontoparietal and limbic fMRI networks previously activated by tasks related to memory, theory of mind, and social cognition. Disruptive maturation was also characteristic of subcortical structures.

We hypothesized that the disruptive pattern of changes in macroscopic FC, measured by fMRI, was reflective of changes in microscopic, synaptic connectivity in association cortical and subcortical brain systems (2). We explored this hypothesis by comparing the fMRI map of MI with prior brain maps of structural, genomic, and metabolic parameters of adolescent development.

PET has been used to map oxidative metabolism of glucose and nonoxidative metabolism of glucose in the presence of oxygen: aerobic glycolysis (AG). AG is thought to generate energy specifically for brain developmental processes, and PET measurements of GI demonstrated that association cortex has sustained AG throughout adolescence to early adulthood (14, 28) (whereas primary cortical areas had relatively low AG after late childhood [14, 28]). We found that GI was highly correlated with MI. Association cortical and subcortical regions with MI < 0 had GI > 0, whereas motor and sensory cortical areas with MI > 0 had GI < 0. This result was corroborated by the significant spatial correlation between a prior map of expression of AG-related genes and the fMRI map of MI. Disruptively developing brain regions had higher levels of AG-related genes than conservatively developing regions. We regard these convergent results as indicating that disruptive adolescent development of fMRI connectivity represents a metabolically expensive process of remodeling in association cortex and subcortical structures.

We also found significant correspondence between the fMRI map of MI and the map of cortical shrinkage derived from structural MRI data in the same sample. Cortical shrinkage is the most well-replicated result in MRI studies of adolescent brain development and has been mechanistically explained as a marker of synaptic pruning and/or intracortical myelination (1). Another structural measure of developmental activity was provided by a prior map of postnatal expansion of cortical surface area (27). Association cortex has both greater surface area expansion and more disruptive development of FC. We regard these results as convergently indicating that disruption of FC between regions is colocated with cortical systems that are most structurally active in their adolescent development.

Finally, we used metaanalysis of existing task-related fMRI data to identify cognitive processes that activated cortical areas coinciding with the 2 modes of adolescent brain development. Conservative systems were activated by sensory and motor functions that would normally have been operational since early childhood. Disruptive systems were activated by a range of “higher-order” functions, such as working memory, theory of mind, and autobiographical memory, which are later maturing social and cognitive processes.

These results generate the hypothesis that disruptive maturation of FC drives the emergence of more sophisticated socializing, mentalizing, and executive skills as young people grow into independent adults. Moreover, they support the corollary hypothesis that psychiatric disorders or subclinical psychopathology could arise in young people from atypical maturation of association cortico-subcortical circuits (32–34).

### Methodological Issues.

Strengths of the study include the accelerated longitudinal design and the balanced sample of healthy young people stratified by age and sex. Limitations include colocation of adolescent fMRI maps with prior maps of gene expression measured post mortem in adults, lack of simultaneously measured cognitive or behavioral data, and insufficient resolution of 3 Tesla (3T) MRI to measure the multiple functionally specialized subnuclei comprising subcortical nodes.

Concerning the crucial factor of in-scanner head motion (15–17), for our main analysis, we processed multiecho fMRI time series with ME-ICA in an effort to disambiguate BOLD components from nonneuronal sources of fMRI dynamics (19, 20). This denoising step alone was not sufficient (23), and therefore, we used regression to further correct FC for linear dependence on head motion (FD regression) (21, 22). Data preprocessed by this pipeline passed standard quality control criteria for head movement impact on FC (*SI Appendix*, Fig. S2). To assess the robustness of our results to this choice of movement correction pipeline, we conducted 2 major sensitivity analyses of a low-motion dataset and of the whole dataset after motion correction by GSR. The results were not identical across main, low-motion, and GSR analyses, but there are many possible factors, besides uncorrected or corrected effects of head motion, that could contribute to observed differences (e.g., the smaller sample size and shorter length of fMRI time series available for the low-motion analysis). However, it is reassuring that estimates of MI, baseline FC, and adolescent change in FC were strongly correlated between different movement correction pipelines (*SI Appendix*, Figs. S36 and S37), and on this basis, key results of our main analysis were replicated in the low-motion subset of scans and in the GSR-corrected scans; *SI Appendix* contains details.

### Conclusion.

Disruptive change in FC between association cortex and subcortical nuclei is likely reflective of a metabolically expensive process of human brain development in adolescence.

## Materials and Methods

### Participants.

A demographically balanced cohort of 298 healthy adolescents (151 females) aged 14 to 26 y, scanned a total of 520 times, was included in this study. There were approximately equal numbers of male and female participants (∼60) in each of 5 age-defined strata at baseline: 14 to 15, 16 to 17, 18 to 19, 20 to 21, and 22 to 24 y inclusive. The study was approved by the National Research Ethics Service and conducted in accordance with NHS research governance standards. Participants aged 16 or older gave informed consent; younger participants gave informed assent and had parental consent.

### MRI Acquisition and Preprocessing.

Scanning was performed at 3 sites, all operating identical 3T MRI systems (Magnetom TIM Trio; Siemens Healthcare; VB17 software). Resting-state fMRI data were acquired using a multiecho echoplanar imaging (EPI) sequence (18): 263 volumes; repetition time (TR) = 2.42 s; GeneRalized Autocalibrating Partial Parallel Acquisition (GRAPPA) with acceleration = 2; matrix size = 64 × 64 × 34; field of view (FOV) = 240 × 240 mm; in-plane resolution = 3.75 × 3.75 mm; slice thickness = 3.75 mm with 10% gap, 34 oblique slices; bandwidth = 2,368 Hz per pixel; echo time (TE) = 13, 30.55, 48.1 ms.

For fMRI preprocessing, we used ME-ICA (19, 20) to identify the sources of variance in the fMRI time series that scaled linearly with TE and could therefore be confidently regarded as BOLD signal. Other sources of fMRI variance that were not BOLD-related and therefore did not scale with TE were identified by ME-ICA and discarded. The retained components, representing BOLD contrast, were recomposed to generate a broadband denoised fMRI time series at each voxel (35). This was bandpass filtered by the discrete wavelet transform (Daubechies 4 wavelet), resulting in a BOLD signal in the frequency range 0.025–0.111 Hz. Geometric realignment of scan volumes was used to estimate 6 motion parameters (3 translation, 3 rotation), from which we derived estimates of volume-to-volume head motion or FD (15). Mean FD was used as a measure of head movement in each scan session.

### Parcellation and FC Estimation.

fMRI data were parcellated by a prior cortical template into 360 bilaterally symmetric regions (36) as well as 16 subcortical regions from FreeSurfer software (37), yielding a total of 376 regions. Regional fMRI time series were estimated by averaging over all voxels in each parcel; 30 cortical regions (near frontal and temporal poles) were excluded due to low regional mean signal, defined by a low *Z* score of mean signal intensity (Z<−1.96) in at least one scan. For sensitivity analyses, we used an alternative parcellation of cortex into 308 parcels of approximately equal surface area (∼5 cm^2^) (38, 39) (*SI Appendix*).

FC matrices were estimated for each scan using Pearson’s correlations between all pairs of regional time series. Residual dependence of FC on head movement (mean FD) was corrected using linear regression (*Head Movement* and *SI Appendix*, Fig. S2 contain details). Age-related change in FC was modeled using linear mixed effect models that included age as the main fixed effect of interest, sex and scanner site as fixed effect covariates, and a subject-specific intercept as a random effect (*SI Appendix* contains further details).

### Data and Code.

The data used for analyses are available at https://doi.org/10.6084/m9.figshare.11551602 (40), and the code can be found at https://github.com/frantisekvasa/functional_network_development (41).

## Supplementary Material

Supplementary File
