# Supplementary material for: Conservative and disruptive modes of adolescent change in human brain functional connectivity
Source: Proc Natl Acad Sci U S A. 2020 Jan 28;117(6):3248–53. doi: 10.1073/pnas.1906144117 (PMC7022153; doi:10.1073/pnas.1906144117)
Supplement: Supplementary File [file pnas.1906144117.sapp.pdf]

PNAS
www.pnas.org

Supplementary Information for

Conservative and disruptive modes of adolescent change in brain functional connectivity

František Váša, Rafael Romero-Garcia, Manfred G. Kitzbichler, Jakob Seidlitz, Kirstie J. Whitaker, Matilde M. Vaghi, Prantik Kundu, Ameera X. Patel, Peter Fonagy, Raymond J. Dolan, Peter B. Jones, Ian M. Goodyer, the NSPN Consortium, Petra E. Vértes*, Edward T. Bullmore*

František Váša.
E-mail: fdv247@gmail.com

* PEV and ETB are joint last authors

Contents

Data **3**
Participants . . . 3
MRI acquisition and pre-processing . . . 3
Parcellation and network construction . . . 3
Exclusion criteria and final sample . . . 4

Effects of motion on functional connectivity **4**

Maturation of functional connectivity **4**
Global maturation of functional connectivity . . . 4
Nodal maturation of functional connectivity . . . 5
Edge-wise maturation of functional connectivity . . . 5

Spatial permutation "spin" test for comparison of cortical maps **6**
Use of the spatial permutation test at the edge level . . . 6

Contextualising findings **6**
von Economo and Yeo atlases . . . 6
Neurosynth . . . 6
Maps of cortical development from the NSPN study . . . 6
Independent cortical maps . . . 7
Gene expression analyses . . . 7

Sensitivity analyses **7**
Split-half analysis . . . 7
Separate estimation of MI using cortico-cortical and subcortico-subcortical edges . . . 7
Alternative parcellation . . . 7
Cross-sectional data . . . 8
Single-scanner data . . . 8
Low-motion subset of data . . . 8
Global signal regression (GSR) . . . 8

Comparison of main results between denoising pipelines **8**

Neuroscience in Psychiatry Network (NSPN) Consortium author list **52**

www.pnas.org/cgi/doi/10.1073/pnas.1906144117

List of Figures

S1 Distribution of scans as a function of age . . . 10
S2 Effects of head motion on functional connectivity . . . 11
S3 Global trajectories of the correlation distribution . . . 12
S4 Maturation of subcortico-cortical and subcortico-subcortical functional connectivity . . . 13
S5 Statistical significance of conservative and disruptive modes . . . 14
S6 Maturation of functional connectivity at the edge level . . . 15
S7 Relationship of changes in FC to Euclidean distance . . . 16
S8 Inspecting edge-wise trajectories for potential presence of non-linearities . . . 17
S9 Relationships between the maturational index and additional cortical maps . . . 18
S10 Maturational Index is robust to random half-splitting of the data . . . 19
S11 Maturational Index is robust to separate estimation using cortico-cortical and subcortico-subcortical edges . . . 20

DK-sub parcellation **21**
S12 Replication of main Fig. 1 in the DK-sub parcellation . . . 21
S13 Replication of main Fig. 2 in the DK-sub parcellation . . . 22
S14 Replication of main Fig. 3 in the DK-sub parcellation . . . 23
S15 Replication of main Fig. 4 in the DK-sub parcellation . . . 24

Cross-sectional data **25**
S16 Replication of main Fig. 1 in cross-sectional data . . . 25
S17 Replication of main Fig. 2 in cross-sectional data . . . 26
S18 Replication of main Fig. 3 in cross-sectional data . . . 27
S19 Replication of main Fig. 4 in cross-sectional data . . . 28

Single-scanner data **29**
S20 Replication of main Fig. 1 in data from a single scanner . . . 29
S21 Replication of main Fig. 2 in data from a single scanner . . . 30
S22 Replication of main Fig. 3 in data from a single scanner . . . 31
S23 Replication of main Fig. 4 in data from a single scanner . . . 32

Low-motion subset of data **33**
S24 Effects of head motion on FC in a low-motion subset of data . . . 33
S25 Replication of main Fig. 1 in a low-motion subset of data . . . 34
S26 Replication of main Fig. 2 in a low-motion subset of data . . . 35
S27 Replication of main Fig. 3 in a low-motion subset of data . . . 36
S28 Replication of main Fig. 4 in a low-motion subset of data . . . 37

Data processed with global signal regression (GSR) **38**
S29 Effects of head motion on FC in data processed with GSR . . . 38
S30 Replication of main Fig. 1 in data processed with GSR . . . 39
S31 Replication of main Fig. 2 in data processed with GSR . . . 40
S32 Replication of main Fig. 3 in data processed with GSR . . . 41
S33 Replication of main Fig. 4 in data processed with GSR . . . 42
S34 Replication of SI Fig. S3 in data processed with GSR . . . 43
S35 Replication of SI Fig. S6 in data processed with GSR . . . 44

Comparison of results across denoising pipelines **45**
S36 Comparison of regional and edge FC_{14} and ΔFC_{14-26} across denoising pipelines . . . 45
S37 Comparison of Maturational Index across denoising pipelines . . . 46

List of Tables

S1 Summary of adolescent development of whole-brain resting-state functional networks . . . 48
S2 Summary of adolescent development of subcortical resting-state functional networks . . . 49
S3 Maturation of subcortico-cortical FC . . . 50
S4 Relationships of maturational index with a range of cortical maps . . . 51

Supporting Information Text

Data

Participants. A demographically balanced cohort of 306 adolescents (153 females) aged 14-26 years was considered for inclusion in this study. Of these, 303 were scanned at "baseline", with approximately 60 participants in each of 5 age-defined strata: 14-15 years inclusive, 16-17 years, 18-19 years, 20-21 years and 22-26 years. Subsequently, 29 participants were scanned approximately 6 months later, and 224 participants were scanned approximately a year later. Thus, a total of 556 scans were acquired. Following exclusion of certain scans, 520 scans were retained; see below and Fig. S1.

Participants provided informed written consent for each aspect of the study, and parental consent was obtained for those aged 14–15 years. None of the included participants were currently being treated for a psychiatric disorder or for drug or alcohol dependence; had a current or past history of neurological disorders or trauma; or had a learning disability. The study was ethically approved by the National Research Ethics Service and was conducted in accordance with NHS research governance standards.

MRI acquisition and pre-processing. Scanning took place at three sites, all operating identical 3T MRI systems (Magnetom TIM Trio, Siemens Healthcare, VB17 software version) with standard 32-channel radio-frequency (RF) receive head coil and RF body coil for transmission. Resting-state fMRI data were acquired using a multi-echo echoplanar imaging (ME-EPI) sequence with online reconstruction (1): 263 volumes; repetition time (TR) = 2.42 s; GRAPPA with acceleration factor = 2; flip angle = 90°; matrix size = 64 x 64 x 34; FOV = 240 x 240 mm; in-plane resolution = 3.75 x 3.75 mm; slice thickness = 3.75 mm with 10% gap, sequential slice acquisition, 34 oblique slices; bandwidth = 2,368 Hz/pixel; echo time (TE) = 13, 30.55 and 48.1 ms.

Following processing of individual structural scans using FreeSurfer v5.3.0 (including skull-stripping, segmentation of cortical grey and white matter and reconstruction of the cortical surface and grey-white matter boundary) (2), all scans were stringently quality controlled by re-running the reconstruction algorithm after the addition of control points and white matter edits (as described previously; 3, 4).

The pre-processing for ME-ICA analysis was done using AFNI. Volumes acquired during steady-state equilibration (15 s) were omitted. The data of the middle TE were used to compute parameters of motion correction and anatomical-functional coregistration, and the first volume after equilibration was used as the base EPI image. Matrices for de-obliquing and six-parameter rigid body motion correction were computed. Then, 12-parameter affine anatomical-functional coregistration was computed using the LPC cost functional (5), using the EPI base image as the LPC weight mask. Matrices for de-obliquing, motion correction, and anatomical-functional coregistration were combined into a single alignment matrix using the concatenation approach from the AFNI tool *align_epi_anat.py*. The dataset of each TE was then slice-time corrected and spatially aligned through application of the alignment matrix. Coregistration of structural and functional scans was visually assessed.

For pre-processing of functional scans, we used multi-echo independent component analysis (ME-ICA; 6, 7) to identify the sources of variance in the fMRI time series that scaled linearly with TE and could therefore be confidently regarded as indicative of BOLD contrast. Other sources of fMRI variance, such as head movement, which were not BOLD-dependent and therefore did not scale with TE, were identified by ME-ICA and discarded. The retained independent components, representing BOLD contrast, were optimally recomposed to generate a broadband denoised fMRI time series at each voxel (8). This was bandpass filtered by the discrete wavelet transform (Daubechies 4 wavelet), resulting in a BOLD signal oscillating in the frequency range 0.025-0.111 Hz (wavelet scales 2 and 3).

During processing, re-alignment of scans was used to estimate 6 motion parameters for each participant (3 translation parameters and 3 rotation parameters). Subsequently, these were used to calculate an overall estimate of motion - the framewise displacement (FD), defined as the sum of the absolute derivatives of the six motion parameters, following conversion of rotational parameters to distances by computing the arc length displacement on the surface of a sphere with radius 50 mm (as in (9) and (10)):

$$FD_t = \sum_d \left| d_{(t-1)} - d_t \right| + 50 \cdot \frac{\pi}{180} \cdot \sum_r \left| r_{(t-1)} - r_t \right| \quad [1]$$

where d denotes translation distances $\{x,y,z\}$, and r denotes rotation angles $\{\alpha,\beta,\gamma\}$. For each participant, mean FD was calculated by averaging the FD time series.

Parcellation and network construction. Each participant's cortex was parcellated using two parcellations: a recent multi-modal parcellation into 360 bilaterally symmetric regions based on data from the Human Connectome Project (11), as well as a sub-parcellation of the Desikan-Kiliany anatomical atlas (12) into 308 parcels of approximately equal surface area (∼5cm^2; 13). Additionally, 16 subcortical regions were provided by FreeSurfer software (14), and included bilateral pairs of the following regions: thalamus, caudate, putamen, pallidum, hippocampus, amygdala, nucleus accumbens and the ventral diencephalon. Thus, the combination of each of the two cortical parcellations with the subcortical regions yielded parcellations of cortex and subcortex into 376 regions (henceforth referred to as the HCP parcellation) and 324 regions (henceforth referred to as the DK-sub parcellation) respectively (before exclusion of "dropout" regions; see below).

Surface parcellations generated by Freesurfer were transformed into the volume using Freesurfer command *mri_aparc2aseg*. Subsequently, regional BOLD time series were estimated by averaging time series over all voxels in each volumetric parcel.

Some regions (particularly near the frontal and temporal poles) were excluded because of low regional mean signal, defined by a low Z-score of mean signal intensity in at least one participant (Z < -1.96); this resulted in the exclusion of 30 cortical regions for the HCP parcellation, and 26 regions for the DK-sub parcellation, primarily in inferior temporal and pre-frontal cortex. Thus, the final number of regions (both cortical and subcortical) used for analyses was 346 in the HCP parcellation, and 298 in the DK-sub parcellation.

Finally, FC matrices were constructed by cross-correlating the BOLD time series between pairs of the retained regions (using Pearson's correlation).

Exclusion criteria and final sample. A total of 36 scans were excluded. Of these: 17 scans were excluded due to high in-scanner motion (defined as mean FD > 0.3 mm or maximum FD > 1.3 mm), 9 due to coregistration errors, 7 due to a lack of convergence of the ME-ICA algorithm, 2 due to parcellation errors, 1 due to extensive dropout (defined above).

Following quality control and participant exclusion, 298 adolescents (151 females), scanned a total of 520 times, were included in the study. Of these, 298 scans were acquired at "baseline", 27 were acquired approximately 6 months later, and 195 were acquired approximately a year later (Fig. S1). Within the final included sample, 95 participants were scanned once, 184 participants were scanned twice, and 15 participants were scanned three times. Thus, the study included 520 unique FC matrices capturing pair-wise FC of 346 (HCP) and 298 (DK-sub) cortical and subcortical regions.

Effects of motion on functional connectivity

We first verified whether participants' overall motion (quantified using time-averaged FD) changes as a function of age (using linear mixed effects models; see details below). Moreover, to evaluate potential residual effects of motion on FC, we calculated the correlation between FC at each edge and participants' motion (across participants). Subsequently, to assess whether motion affects FC in a distance-dependent manner, we correlated the upper triangular part of this matrix (of correlations between FC and motion) to the upper triangular part of a matrix of Euclidean distances between the centroids of all regions, as in (15).

We found that the average participant motion (as measured by FD) did not change with age ($t(219) = 1.27, P = 0.20$; Fig. S2A). However we did find residual signatures of motion in our data, including relationships between average participant motion and FC at several spatial scales, and a weak distance-dependence of the relationship between FC and motion at each edge (Fig. S2B,C). In the present work, we have removed these residual effects using regression, as has been applied previously (16) (Fig. S2D). Following regression of mean FD from edge-wise FC, mean FC was not related to mean FD across participants ($t(221) = -1.1, P = 0.27$; Fig. S2F).

Additionally, to demonstrate that our results are not biased by the specific processing methods we have used, we repeated analyses in a subset of data showing no discernible motion, as well as in data preprocessed using global signal regression (GSR); for details, see *Sensitivity analyses* section below, Fig. S24-S37 and Table S4.

Maturation of functional connectivity

Maturation of FC as a function of age was modelled using linear mixed effect models. These models included age as the main fixed effect of interest, sex and scanner site as fixed effect covariates, as well as a subject-specific intercept as a random effect, as follows:

$$FC = \beta_{age} \cdot age + \beta_{sex} \cdot sex + \beta_{site} \cdot site + \gamma_{subj} \cdot (1|subj) + \epsilon \qquad [2]$$

where FC refers to functional connectivity (at one of several possible levels of spatial resolution; see below); β refers to coefficients for the fixed effects, γ refers to coefficients for random effects and ϵ represents the residual error. We further explored inclusion of an interaction term between age and sex, as well as intra-cranial volume (ICV) as additional covariates. These models presented poorer fits (as indicated by increased AIC and BIC) than models without these terms fitted to the same data. Therefore, we focused on fixed effects of age and sex only.

Additionally, we fitted locally adaptive mixed effect smoothing splines (generalized additive mixed models) to regional trajectories, better suited than quadratic models at modelling non-linear trajectories (17). Specifically, we used the *gamm* function in R, with effect of age modelled as a weighted sum of 10 cubic b-splines with knots placed at quantiles of the data and smoothing optimized using restricted maximum likelihood (REML) (18). We inspected for evidence of non-linearity using the degrees of freedom (df) of the smoothing splines. However, we found no substantial evidence of strong non-linearities, and thus focused on linear trajectories (see Fig. S8).

Linear mixed effect models were fitted at multiple spatial scales: (i) globally, at the level of the correlation distribution, (ii) regionally, using average nodal FC and (iii) edge-wise.

Global maturation of functional connectivity. To evaluate maturational shifts in the functional correlation distribution, we first summarised each individual distribution using its four moments: the mean, variance, skewness and kurtosis, fitting linear models as a function of age to each. Further, to more precisely characterise potential local age-shifts in the correlation distribution, we fitted linear models to the value of correlation at each percentile of the distribution, and compared the effect sizes across percentiles.

The correlation between fMRI time series for a pair of cortical and/or subcortical regions was generally positive indicating FC through coherent in-phase low frequency BOLD oscillations. The global parameters of the correlation distributions, estimated over all ∼60,000 edges in each correlation matrix, shifted over adolescence. Between 14 and 26 years, there was a weak but significant trend for the average or mean correlation to increase ($t(221) = 2.3$, $P = 0.023$). There were also stronger age-related effects on the higher-order parameters of the correlation distribution: variance or spread increased ($t(221) = 4.9$, $P = 1.7{\cdot}10^{-6}$)); skewness decreased ($t(221) = -2.1$, $P = 0.040$)); and kurtosis or peakiness decreased ($t(221) = -4.4$, $P = 2.0{\cdot}10^{-5}$) (Fig. S3A). We also characterised changes in the correlation distribution more precisely by fitting linear models to estimate the effect of age on the value of correlation at each percentile. Higher percentiles of the correlation distribution demonstrated the most rapid rates of further increase in positive correlation over the course of adolescence (Fig. S3B).

Nodal maturation of functional connectivity. We next focused on individual regions using node strength. We calculated node strength separately over subsets of cortical edges (between the 330 cortical regions and all 346 regions) and subcortical edges (between the 16 subcortical regions and all 346 regions). We characterised regional development of FC by fitting linear models of changes in node strength as a function of age. In addition to the effect size of changes as a function of age (ΔFC_{14-26}), we extracted predicted FC at age 14 years (FC_{14}). Maps of cortico-cortical and cortico-subcortical connectivity are reported in main Fig. 1B. For maps of and subcortico-cortical and subcortico-subcortical connectivity, see Fig. S4.

Moreover, we constructed maps of subcortico-cortical maturation of FC for each of the eight bilateral pairs of subcortical regions listed in section *Parcellation and network construction* above. Maps of the baseline FC (FC_{14}) and development of FC (ΔFC_{14-26}) to each cortical node were obtained by fitting linear models to the average of the two edges between each bilateral pair of subcortical regions and each cortical region (main Fig. 1C).

While many of the subcortico-cortical age-related changes were statistically significant at an uncorrected probability threshold ($P < 0.05$), only the amygdala and the hippocampus had significantly increased connectivity to some cortical areas when controlling the false discovery rate (FDR < 0.05; Table S3).

Edge-wise maturation of functional connectivity. Finally, the most fine-grained description of FC development was obtained by fitting linear models as a function of age to each edge (Pearson's correlation) in the FC matrix. This led to matrices of the edge-wise effect sizes of age (ΔFC_{14-26}), as well as edge-wise baseline (FC_{14}). These matrices were explored in several ways.

Maturational Index. As described in the main text, we evaluated at each node the relationships (Spearman's ρ) between corresponding edge-wise parameters: FC_{14} and ΔFC_{14-26}, which we term "Maturational Index" (MI). By being derived from connection-wise mixed-effects linear models, the MI describes connection-level effects; however (by definition of the MI as a correlation between edge-wise FC_{14} and ΔFC_{14-26} *at each node*) these results are presented at the regional or nodal level.

Edge-wise maturation within and between cytoarchitectonic classes and functional networks. To further illustrate changes at the connection level, we visualised edge-wise matrices of FC_{14} and ΔFC_{14-26} within (a) von Economo cytoarchitectonic classes (19), and (b) functional MRI networks previously derived by Yeo et al. (20). For details regarding mapping of these atlases to the cortical parcellations used, see section *Contextualising findings* below. We plotted summary matrices of FC_{14} and ΔFC_{14-26} both at the level of individual edges (with nodes sorted by decreasing value within network), and at the average within- and between-network level, to provide a comprehensive visual assessment of network-wise edge-level effects (Fig. S6).

Panels in Fig. S6 complement the maturational index to illustrate differences in adolescent development of functional connectivity within and between (i) primary cortical subnetworks, and (ii) association cortical and subcortical subnetworks. Generally, (i) in "lower-order" primary classes and networks, high baseline connectivity (FC_{14}) within and between subnetworks corresponds to high adolescent rates of increase in connectivity (ΔFC_{14-26}). Conversely, (ii) "higher-order" association-cortical and subcortical subnetworks undergo a more diverse combination of increases and decreases in connectivity over adolescence (as apparent from both positive and negative edge-wise entries in ΔFC_{14-26} matrices), which average to low values of overall apparent change. The MI further illustrates that this association-cortical and subcortical combination of increases and decreases in FC is not random, but rather patterned according to edge-wise baseline connectivity.

Relationship of edge-wise maturation to Euclidean distance. We next inspected potential effects of the distance of functional connections on their maturation, by evaluating Spearman's correlation between the upper triangular parts of the matrices of Euclidean distance (between pairs of region centroids) and ΔFC_{14-26}.

We found no evidence of such a relationship; in data reported in the main text (Spearman's $\rho = 0.0024$, $P = 0.56$; Fig. S7), in a low-motion subset data (Spearman's $\rho = 0.013$, $P = 0.00098$) or in data processed using GSR (Spearman's $\rho = 0.0045$, $P = 0.27$). This indicates that adolescent maturation of FC was not greater for longer (or shorter) distance connections.

Fitting of smoothing splines to edge-wise FC trajectories. Finally, to inspect maturational trajectories for the potential presence of non-linearities, we fitted locally adaptive mixed effect smoothing splines to edge-wise data. Subsequently, we extracted the effective degrees of freedom (df) of the smoothing splines ($df = 2$ corresponds to a linear trajectory).

Most trajectories were linear: of all edges (in the HCP parcellation), 71.7% trajectories had $df < 2.1$, and 80.0% had $df < 2.5$ (Fig. S8A). The cortical distribution of average nodal df (averaged across all of a node's edges) showed that regions with $df > 2.5$ were scattered across cortex, and not systematically located within specific anatomical or functional areas (Fig. S8B). Finally, the maturational index (MI) showed a weak relationship with average nodal df (Spearman's $\rho = -0.11$, $P = 0.045$, $P_{\text{spin}} = 0.016$), with a tendency of "disruptive" regions (with MI < 0) to show more (weakly) non-linear trajectories (Fig. S8C).

This is in line with prior work showing adolescent non-linearities in association-cortical trajectories of both cerebral perfusion (21), and structural covariance (4). However, given that most regions showed approximately linear trajectories, we focused on results extracted using linear mixed effect models, and on linear rates of change (ΔFC_{14-26}), in main analyses.

Spatial permutation "spin" test for comparison of cortical maps

When relating pairs of regional cortical maps to each other across regions, non-independence among regions (due to spatial auto-correlation amongst neighbouring parcels) should be taken into account. Here, we used an updated version of a regional spherical permutation test implemented in Váša et al. (4), following earlier implementation of such tests at the vertex level (22, 23).

The spatial permutation test was implemented in the following manner: First, we obtained the coordinates of the centroids of each of our cortical regions on the FreeSurfer spherical projection of the parcellation. We next generated random rotation matrices following the procedure described in Lefèvre et al. (24), by (i) generating 3×3 matrices with coefficients following independent standard normal distributions, (ii) applying a QR decomposition, and (iii) keeping the orthogonal matrices Q which are uniformly distributed. This is an improved approach for the generation of random rotations, compared to a prior implementation in Váša et al. (4) (and the initial pre-printed version of this manuscript), where the sphere is rotated about the {x,y,z} axes at three randomly generated angles, which results in non-uniform sampling of the space of spherical permutations; for details, see (25).

Following rotation of the sphere, coordinates of the rotated regions were matched to coordinates of the initial regions, using Euclidean distance and proceeding in a descending order of average Euclidean distance between pairs of regions on the rotated and unrotated spheres (i.e.: starting with the rotated region that is furthest away, on average, from the unrotated regions). The matching then provides a mapping from the set of regions to itself, that allows any regional measure to be permuted while controlling for spatial contiguity and hemispheric symmetry.

P-values for the correlation between two maps were obtained by comparing the empirical value of Spearman's ρ to a null distribution of Spearman correlations, generated by correlating one of the empirical maps to a set of 10'000 spatially permuted versions of the other map. Each analysis correlating values from two cortical maps is reported with both the P-value corresponding to the Spearman correlation (P), as well as a P-value derived from the spherical permutation (P_{spin}).

All P-values (both Spearman P and P_{spin}) relating to comparisons of the Maturational Index with external cortical maps were corrected for a total of 12 multiple comparisons using the false discovery rate (FDR). All FDR-adjusted P-values are reported in Table S4.

Use of the spatial permutation test at the edge level. The spherical permutation test can also be used to rigorously evaluate statistical significance of the correspondence between two sets of a region's edges, as when relating edge-wise FC_{14} and ΔFC_{14-26} to calculate the maturational index at each node. In the edge-wise case, each edge connected to the region being tested is assigned to its other (connecting) node. As with its regional application, the spherical permutation test can only be applied within cortex; at the edge-level, this means that only cortico-cortical and subcortico-cortical edges can be spherically permuted (i.e., the test does not apply to subcortico-subcortical edges).

Contextualising findings

von Economo and Yeo atlases. We mapped the von Economo atlas of 7 cytoarchitectonic classes (19) to our cortical parcellations. For our in-house sub-parcellation of the Desikan-Killiany atlas (13) into 308 cortical regions, we used a manual labeling of Desikan-Killiany regions to von Economo classes, previously described and used in (26). We then mapped this atlas to the HCP parcellation, as follows. We first computed surface overlap (at the vertex level, using CIVET software; 27) between each HCP parcel and each cytoarchitectonic class (based on manual assignment of cytoarchitectonic classes to Desikan-Killiany regions), before assigning each HCP parcel to the class that it overlapped most. Due to symmetry of our original von Economo atlas, we computed overlap across both hemispheres simultaneously, resulting in a symmetric assignment of von Economo classes to HCP parcels. In addition to the seven classes used in (4, 26), we considered an additional "class", consisting of the eight bilateral pairs of subcortical regions included in our anatomical atlas. The Yeo atlas (20) was mapped in a similar manner, except that the original vertex-wise atlas was used.

Neurosynth. To perform the Neurosynth (28) functional meta-analysis, surface maps of the two modes of the maturational index were converted from Freesurfer *fsaverage* to MNI space using barycentric interpolation via the *SurfToSurf* command in AFNI (29). The resultant cortical surface maps were then uploaded to Neurovault and analyzed with the Neurosynth "decoder" function. For each of the two modes, the 35 most relevant cognitive terms were visualised on a word-cloud plot, with size scaled according to their correlation with corresponding meta-analytic maps generated by Neurosynth.

Neurosynth results are intended to contextualize the conservative and disruptive systems defined by adolescent development of functional connectivity. However, these results do not imply that these brain regions or systems are exclusively specialized for these psychological processes.

Maps of structural development from the NSPN study. Maps of cortical rates of thinning (ΔCT; main Fig. 4A) and myelination (ΔMT; Fig. S9A) were derived from cross-sectional structural scans of 297 participants from this study, previously described by Whitaker, Vértes et al. (3). As described in (3), rates of myelination were estimated at approximately 70% cortical depth.

Maturation of structural covariance (SC) was derived from the cortical thickness (CT) data described in (3), following sliding-window procedures described in (4). Specifically, SC was estimated by cross-correlating regional CT measures between all pairs of regions for 9 consecutive and overlapping "windows" comprising ∼60 participants each (obtained by interpolating the 5 age-strata of the NSPN study). Subsequently, rates of change of structural covariance (ΔSC_{CT}; Fig. S9B) were estimated as the slopes of linear models fitted to "windowed" estimates of regional SC, as a function of median age of participants in each window (4).

Independent cortical maps. PET maps of cerebral metabolic rate for glucose (CMRGlu; Fig. S9C), cerebral metabolic rate of oxygen ($CMRO_2$; Fig. S9D), cerebral blood flow (CBF; Fig. S9E) and cerebral blood volume (Fig. S9F) (all originally from (30) and remapped by (31, 32)) were provided via personal correspondence with the research groups that had generated the data. Cortical maps of postnatal surface expansion (main Fig. 4B) and evolutionary surface expansion (Fig. S9G) were made available online by authors (33). Maps of cortical areal scaling (Fig. S9H) were made available by J.S. (who is also a co-author on this study; 32).

We first converted these maps to the *fsaverage6* surface template using standard transformations provided in the HCP Connectome Workbench (v1.2.2) software package (https://www.humanconnectome.org/software/connectome-workbench). Next, we transformed the *fsaverage6*-aligned map to the MNI surface space by a direct surface-to-surface transformation in which values at vertices in the target coordinate matrix were assigned by ones of their nearest neighbor vertices within a 10 mm radius in the source coordinate matrix. To relate these vertex-level maps to our parcel-based results, we first averaged the vertex values within each parcel.

Due to the availability of only the group-level PET maps, the glycolytic index (GI; main Fig. 4C) was calculated at the vertex level by taking the scaled (by 1000) residuals of the group average CMRGlu map regressed on the group average CMRO2 map, as performed at the participant level in (30).

Gene expression analyses. Prior work by Goyal et al. has identified 116 genes whose expression pattern across the cortex was correlated with the extent to which different brain regions rely on aerobic glycolysis (34). Here, we used data from the Allen Human Brain Altas (AHBA) – a transcriptomic dataset created by the Allen Institute for Brain Sciences (http://human.brain-map.org) (35) – to ask whether the mean expression value of these 116 genes also correlates with the maturational index derived from fMRI. The AHBA dataset is based on six donor brains from three Caucasian, two African-American and one Hispanic donors. Their ages were 57, 55, 49, 39, 31 and 24 years. For the results reported in the main manuscript we used the data made available alongside the recent paper (36) which had undergone the following processing steps (as described in the paper): "(i) confirming and updating probe-to-gene annotations using the latest available data; (ii) background filtering, where expression values that do not exceed background are removed; (iii) probe selection, which, for genes indexed by multiple probes, involves selecting a single representative measure to represent the expression of that gene; (iv) sample assignment, where tissue samples from the AHBA are mapped to specific brain regions in an imaging dataset (in this case mapping to the parcels in the HCP parcellations); (v) normalization of expression measures to account for inter-individual differences and outlying values".

We then selected the genes from (34), averaged their expression across cortical brain regions (main Fig. 4D) and correlated this vector with the maturational index. Note that four of the six AHBA donor brains were only sampled in the left hemisphere, due to the presumed similarity of gene expression between hemispheres. For this reason we only use brain regions from the left hemisphere in main text Figure 4D.

In this supplement, we show that the results are unchanged when using a previously published form of the AHBA data preprocessed and matched to the DK sub-parcellation as described in (37) (including slightly different criteria for each of the pre-processing steps described above).

Sensitivity analyses

Split-half analysis. To ensure robustness of our results, we recomputed the MI using edge-wise FC_{14} and ΔFC_{14-26} derived from independent random half-splits of the data. At each iteration, we randomly split the dataset in half, leading to two random sub-samples of 260 scans. We then separately fitted linear mixed-effect models to each half-dataset, to generate separate estimates of FC_{14} and ΔFC_{14-26} from each half. Finally, we correlated these edge-wise parameters *across* the two half-splits at each node, and averaged the two split combinations, to generate the maturational index. We have repeated this random half-split procedure 1000 times, and averaged the results.

The resulting MI map shows slightly decreased values relative to the main text, but is otherwise fully consistent; see Fig. S10.

Separate estimation of MI using cortico-cortical and subcortico-subcortical edges. To account for potential differences in temporal signal-to-noise ratio (tSNR) between cortical and subcortical regions, we re-estimated the maturational index by separately using only cortico-cortical edges, and subcortico-subcortical edges.

The cortical MI map, estimated using only cortico-cortical edges, is fully consistent with the map reported in the main text. The subcortical MI values, estimated using only subcortico-subcortical edges, are consistent with the main text in that subcortical MI values are mostly negative (Fig. S11).

Alternative parcellation. As mentioned in the *Parcellation and network construction* section above, all results and figures presented in the main text for the Human Connectome Project (11) parcellation were replicated with a different parcellation

based on sub-parcellating the Desikan-Kiliany (DK) anatomical atlas (12) into 308 parcels of approximately equal surface area ($\sim$5cm^2; 13), to demonstrate independence of results on the specific atlas used.

In Fig. S12-S15 we show that the key results for all Figures in the main text hold in this alternative parcellation. Similarly, Table S3 recapitulates frequencies of regional increases and decreases in subcortico-cortical FC for both the HCP and DK-sub cortical parcellations. Finally, Table S4 summarises the relationship between maturational index and a range of cortical maps in both parcellations.

Cross-sectional data. Given that our analyses are based on longitudinal fMRI data, there is a potential concern that results could be affected by "regression to the mean": whereby if a value was high at baseline measurement, regression to the mean could drive its decline on follow-up. To exclude the possibility that our results were driven by regression to the mean, we repeated main analyses with the subset of 298 cross-sectional scans from our dataset. The only difference in implementation of code relative to main analyses was use of fixed-effect linear models, by excluding the random-effect term (accounting for repeated measures within participants) from our linear models.

Results of these supplementary analyses were fully consistent with the main text. For details, see Fig. S16-S19 and Table S4.

Single-scanner data. Data used in this study was obtained at three scanner sites. We ran a mixed-effects model to test the distribution of participant's ages across sites, and found an effect of site (F(2,220) = 14.04, $P < 0.0001$). Although we included site as a covariate in all mixed-effect linear models in main analyses, we have performed additional analyses to demonstrate that our results are not driven by site effects. Specifically, we repeated main analyses using only scans from the largest imaging site in our study (Wolfson Brain Imaging Centre [WBIC], Cambridge; 396 scans from 236 participants).

Results of these supplementary analyses were fully consistent with our main results; for details, see Fig. S20-S23 and Table S4.

Low-motion subset of data. To ensure that our results are not biased by the specific processing methods we have used to remove effects of head-motion, including ME-ICA to remove effects of head-motion from BOLD time-series and FD regression to correct for residual dependence of edge-wise FC on mean FD, we repeated main analyses in a subset of data showing no discernible head movement. Specifically, we identified segments of 100 consecutive volumes in 182 scans (from 132 participants) which demonstrate no detectable motion (FD $<$ 0.2 mm at every time-point, based on "detectable motion" criteria from (38)). At TR = 2.42 s, this corresponds to 4.03 min of rs-fMRI data per scan.

We first investigated this subset of "low-motion" data for potential residual effects of motion (Fig. S24). Mean FD ranged from 0.03 to 0.13 mm, and did not vary as a function of participants' ages ($t(47) = 0.29, P = 0.77$; Fig. S24A). The edge-wise correlation between FC and mean FD across participants was low (mean $r = -0.0065$) and showed no substantial relationship to Euclidean distance spanned by edges (Spearman's $\rho = 0.036$; Fig. S24B). Finally, average FC was not related to movement (mean FD) across participants ($t(47) = -0.45, P = 0.65$; Fig. S24C). These results suggest that additional post-processing steps such as FD-regression to control for residual effects of motion are not necessary in this subset of data.

Next, we repeated our main analyses (i.e. main Fig. 1-4) on this subset of scans. Results were broadly qualitatively consistent; for the individual figures, see Fig. S25-S28, for a quantitative comparison of regional and edgewise FC_{14} and ΔFC_{14-26} and Maturational Indices between denoising pipelines, see Fig. S36 and S36 respectively, and for discussion, see section *Comparison of main results between denoising pipelines* below.

Global signal regression (GSR). Global signal regression (GSR) involves regression of the average whole-brain signal (or equivalently the gray-matter signal, which correlates at 0.99 $\pm$ 0.01 with the whole-brain signal; 38) from all voxel-wise or regional time-series, prior to estimation of functional connectivity using correlations. Due to effects such as removal of an unknown mixture of both signal and noise, and zero-centering of the correlation distribution, GSR is perhaps the most controversial processing step in the fMRI literature (see e.g. (39) and references within).

Here, we re-processed the full sample (520 scans) with the addition of GSR. We implemented GSR by regressing average gray-matter signal from all regional time-series, following ME-ICA, and prior to frequency filtering to the frequency band 0.025-0.111 Hz.

We investigated data processed with GSR for potential residual effects of motion (Fig. S29). The (lack of a) relationship between mean FD and age (Fig. S29A) is independent of GSR and thus identical to the main processing pipeline (Fig. S2A). The edge-wise correlation between FC and mean FD across participants was low (mean $r = -0.0039$), and showed a weak relationship to Euclidean distance spanned by edges (Spearman's ρ = -0.10; Fig. S29B). Finally, average FC was weakly related to movement (mean FD) across participants ($t(219) = -4.3$, $P = 2.8 \cdot 10^{-5}$; Fig. S29C).

Next, we repeated our main analyses (i.e. main Fig. 1-4), and several supplementary analyses, on these data. For recreation of main figures with GSR processed data, see Fig. S30-S33; for global and edge-wise changes in FC in data processed with GSR, see S34 and S35; and for a quantitative comparison of (regional and edgewise) FC_{14} and ΔFC_{14-26} as well as Maturational Indices between all three denoising pipelines, see Fig. S36 and S37. For discussion, see section *Comparison of main results between denoising pipelines* below.

Comparison of main results between denoising pipelines

The key results of our main analysis are qualitatively recapitulated by analysis of both the low-motion dataset, and data additionally processed with GSR; in all cases: (i) there are two modes of adolescent change in functional connectivity defined

by positive and negative MI; (ii) conservatively maturing brain systems, defined by MI > 0, are concentrated in primary cortical areas, and disruptively maturing brain systems, defined by MI < 0, are concentrated in subcortical and association cortical areas; and (iii) conservatively and disruptively maturing systems are significantly co-located with prior maps of aerobic glycolysis (AG) and AG-related gene expression.

Fig. S36 demonstrates that nodal and edge-wise functional connectivity at age 14 (FC_{14}), and nodal and edge-wise change in functional connectivity during adolescence (ΔFC_{14-26}), are both strongly positively correlated between main and low-motion analyses. Fig. S37 further demonstrates that MI is strongly positively correlated between main and low-motion analysis for both cortical and subcortical areas.

There are also differences between the results of our main analysis, and low-motion and GSR sensitivity analyses. When comparing results of the main and low-motion analyses, we note that there are many points of difference between these two analyses, besides the difference in pre-processing, that would be expected to contribute to differences in the results: (i) the sample size for the main analysis (N = 520) was substantially larger than the sample size for low-motion analysis (N = 182); (ii) the length of fMRI time series available for estimation of functional connectivity was substantially longer for the main analysis (263 fMRI timepoints) than for the low-motion analysis (100 fMRI timepoints); and (iii) the demographic constitution of the sample for the main analysis was prospectively balanced for age and sex whereas the subsample for low-motion analysis was not. Any or all of these differences between the datasets could have contributed to the observed differences in results, which can therefore not be certainly attributed to differences in pre-processing.

Regarding GSR: given that this step is known to substantially change the distribution of inter-regional correlations, making negative correlations much more frequent and changing the rank order of correlations (39), it is not clear that the differences in results are attributable to GSR having more completely corrected head movement effects than our main processing pipeline. Indeed, there is a small but significant correlation between mean FC and mean FD in the GSR pre-processed data (Fig. S29) whereas there is no significant correlation between FC and FD in either data used for main analyses (Fig. S2) or the low-motion data (Fig. S24).

Taken together, our supplementary analyses results suggest that our choice of motion correction method did not drive our main results and conclusions, of a "conservative" mode of adolescent development present in primary cortex and a "disruptive" mode of adolescent development present in association cortex and subcortex, which are related to externally defined maps of metabolic and genetic processes.

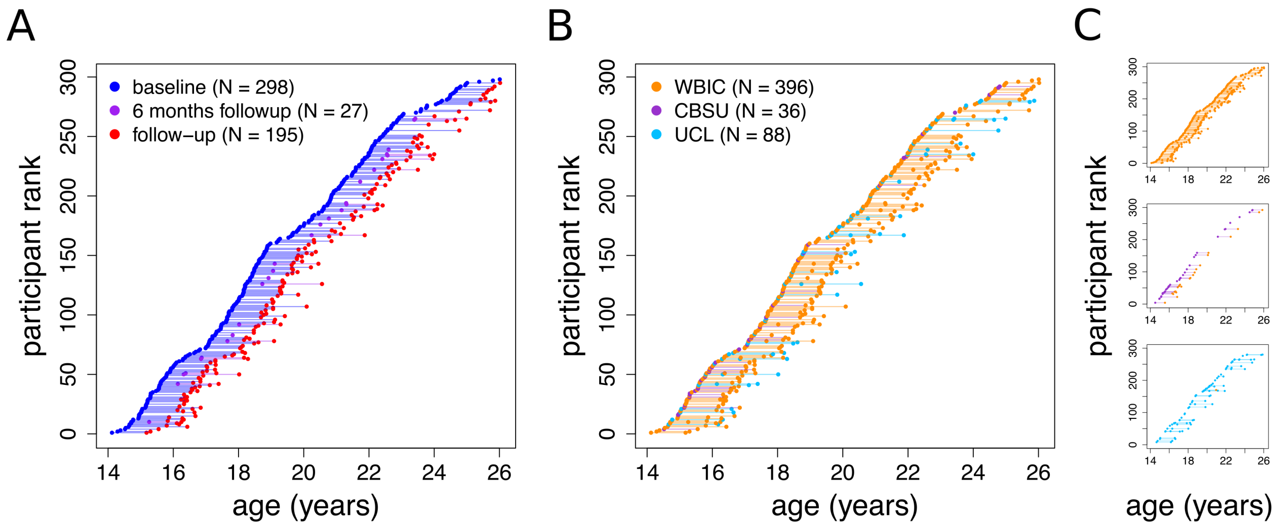

Fig. S1. Distribution of scans as a function of age. Markers correspond to scans retained for inclusion in the study, connecting lines join consecutive scans for the same participant. Participants are ordered by age at first "baseline" scan. A) Markers colored by scan occasion. B) Markers colored by scan site (WBIC: Wolfson Brain Imaging Centre, Cambridge; CBSU: MRC Cognition and Brain Sciences Unit, Cambridge; UCL: University College London, London). C) Site-specific panels, with participants sorted according to baseline scan site.

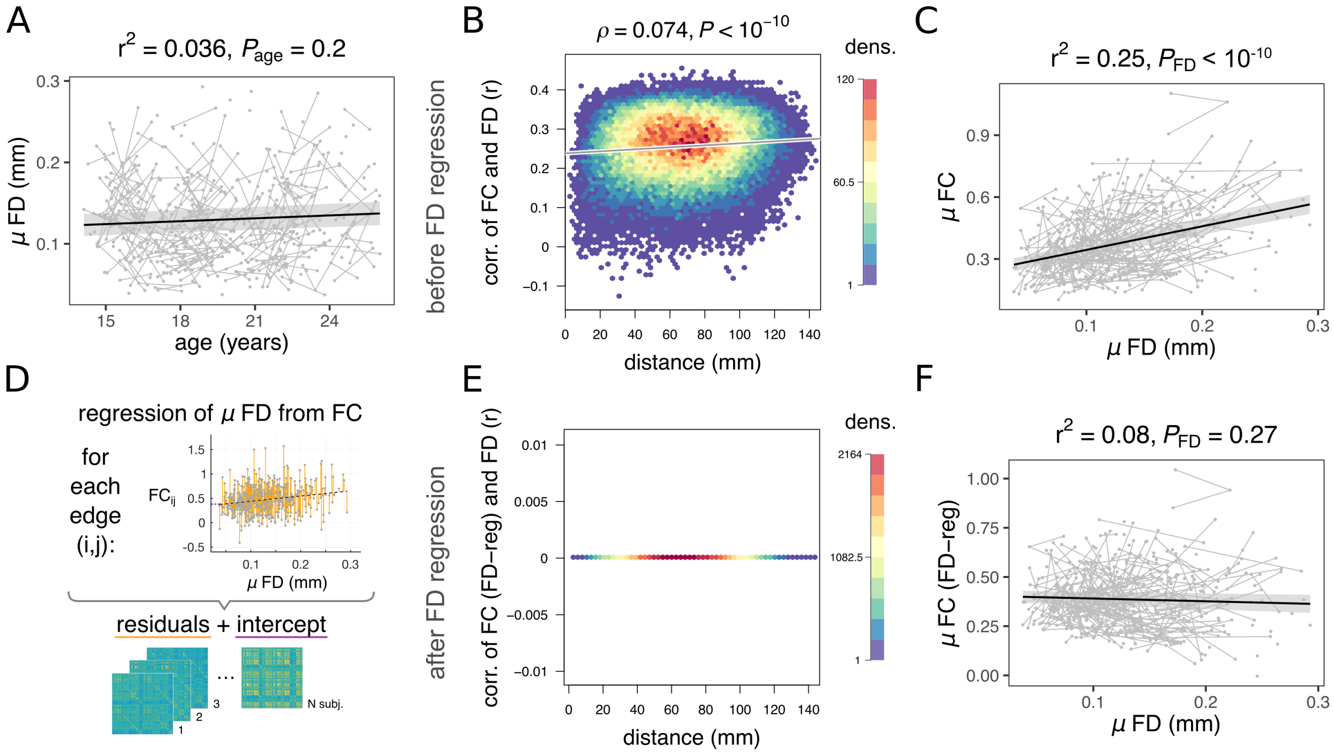

Fig. S2. Effects of head motion on functional connectivity. A) Average participant motion (quantified as mean FD) does not change with age (the pseudo-r^2 for generalized mixed-effect models (GLMMs) includes variance in mean FD explained by age, as well as sex and scanner site). B) The correlation between FC at each edge and the corresponding participant's motion (across participants) shows a weak relationship with the Euclidean distance spanned by edges. Moreover, the average edge-wise correlation between FC and motion is non-zero (as evidenced by a non-zero intercept of the linear regression on the y-axis). C) Mean participant motion is strongly related to mean FC across participants, such that the functional networks of participants who move more are more strongly functionally connected (the pseudo-r^2 for GLMMs includes variance in mean FC explained by mean FD, as well as sex). D) To remove the dependence of FC on motion, mean FD was regressed from each edge; the residuals constitute participant-specific FD-corrected FC, with intercepts retained to maintain the relative importance of edges across the group as well as the interpretability of FC values. Following edge-wise correction of FC for motion, the correlation between FC and motion vanished - both E) at the level of individual edges (by definition), and F) at the whole-brain level (the pseudo-r^2 for GLMMs includes variance in mean FC explained by mean FD, as well as sex). Panels B and E depict 2D-histograms of the underlying dense data, with hexagonal 2D-bins color-coded by the number of edges located within.

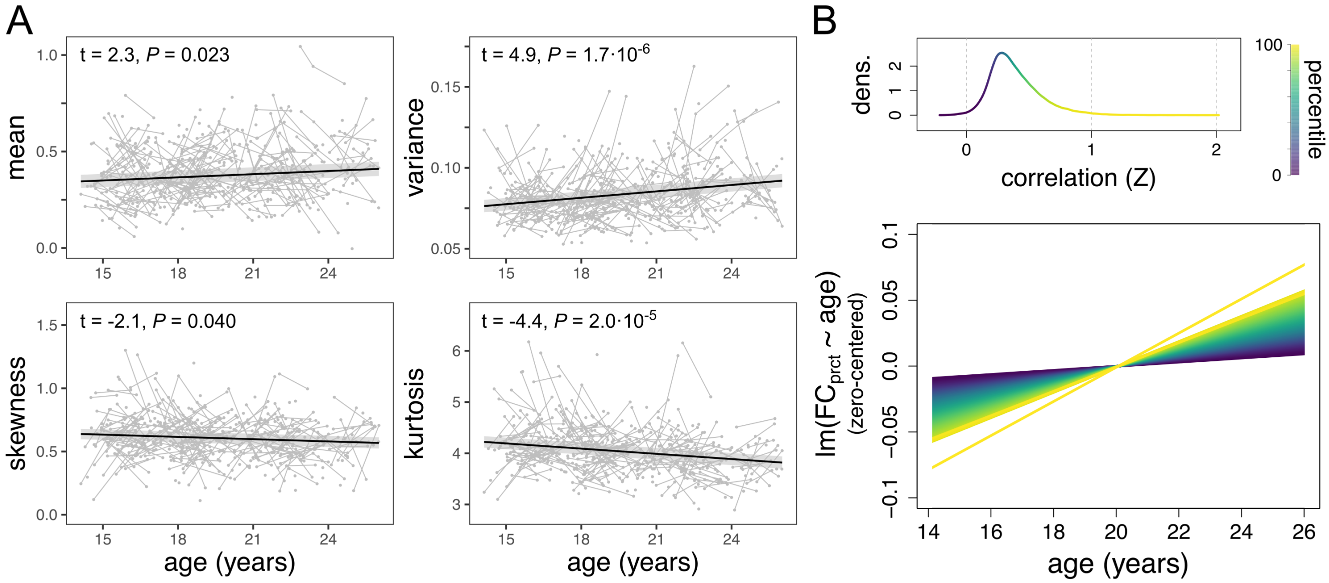

Fig. S3. Global trajectories of the correlation distribution. A) Trajectories of the four moments of the correlation distribution as a function of age: (i) mean, (ii) variance, (iii) skewness and (iv) kurtosis. B) Example distribution, colour-coded by percentile. Higher percentiles of the correlation distribution strengthen faster, as indicated by increasing slopes of linear models fit to values of correlation at increasing percentiles. This is visualised using a plot of all the linear models as a function of age (centered along the y-axis for visual clarity).

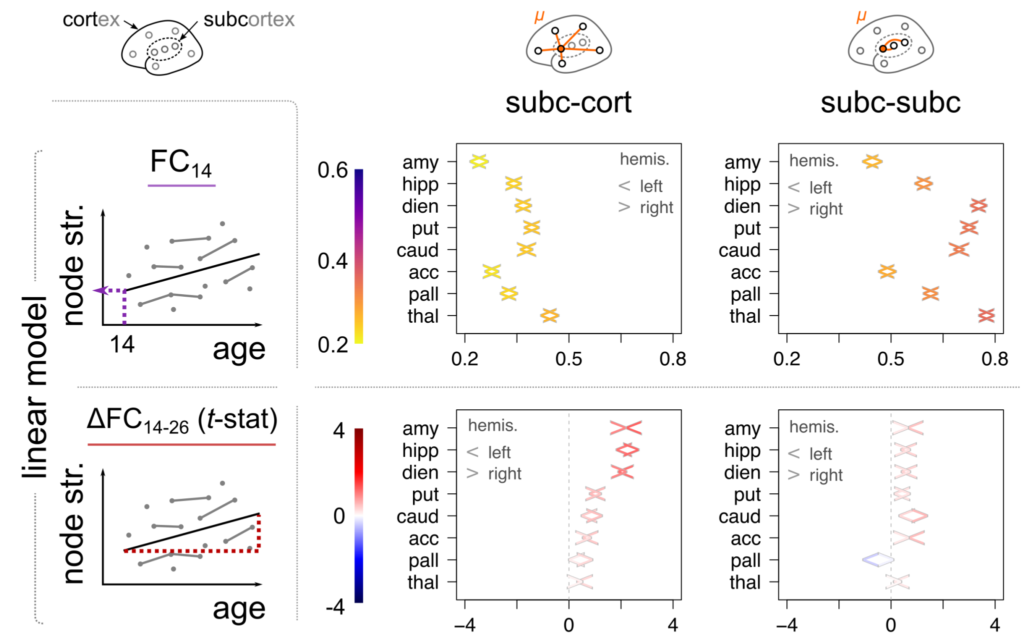

Fig. S4. Maturation of subcortico-cortical and subcortico-subcortical functional connectivity. Parameters of linear models extracted from subcortico-cortical FC (left) and subcortico-subcortical FC (right): FC_{14} (top row) and ΔFC_{14-26} (bottom row). The left/right arrow corresponds to left/right hemisphere. Regions are ordered by decreasing average rate of change in subcortico-cortical connectivity.

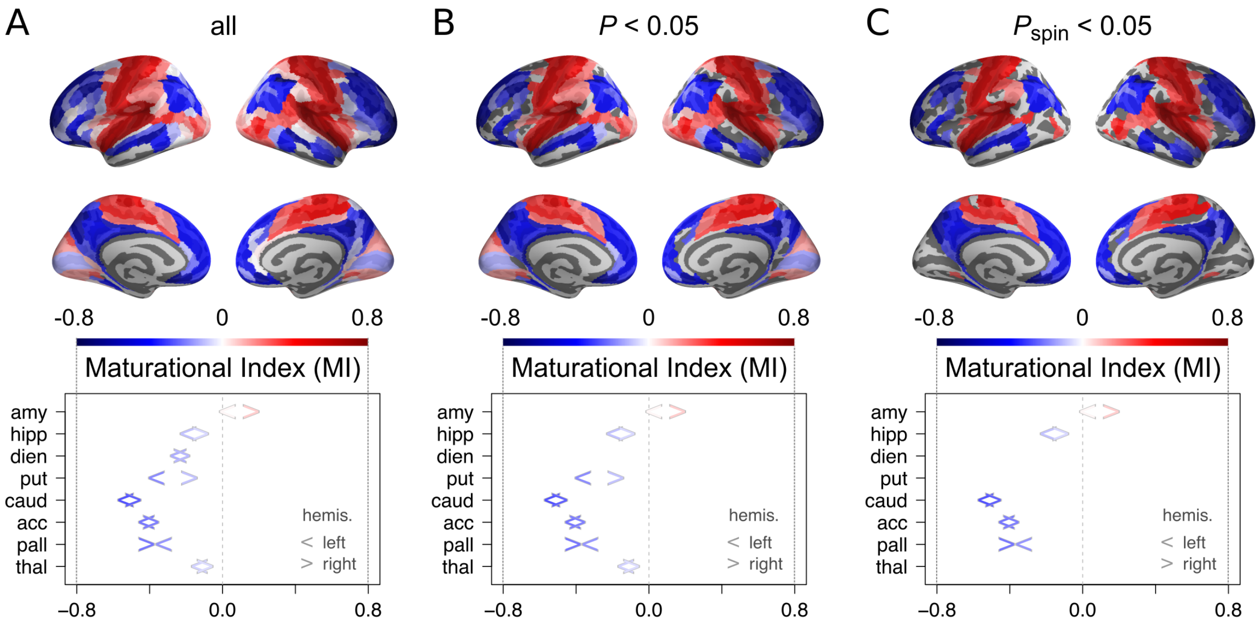

Fig. S5. Statistical significance of conservative and disruptive modes. Conservative and disruptive modes of the maturational index are significantly non-zero by several statistical tests. A) Unthresholded maturational index, presented in main Fig. 2. B) Maturational index thresholded using P-values from Spearman's ρ; only regions with FDR-adjusted $P < 0.05$ are shown. C) Maturational index thresholded using P-values from a spherical permutation test (P_{spin}), controlling for spatial contiguity and hemispheric symmetry of regions; only regions with FDR-adjusted $P_{spin} < 0.05$ are shown. (For further details regarding the sperical permutation test, see section *Spherical permutation "spin" test for comparison of cortical maps* above, and in particular subsection *Use of the spatial permutation test at the edge level.*)

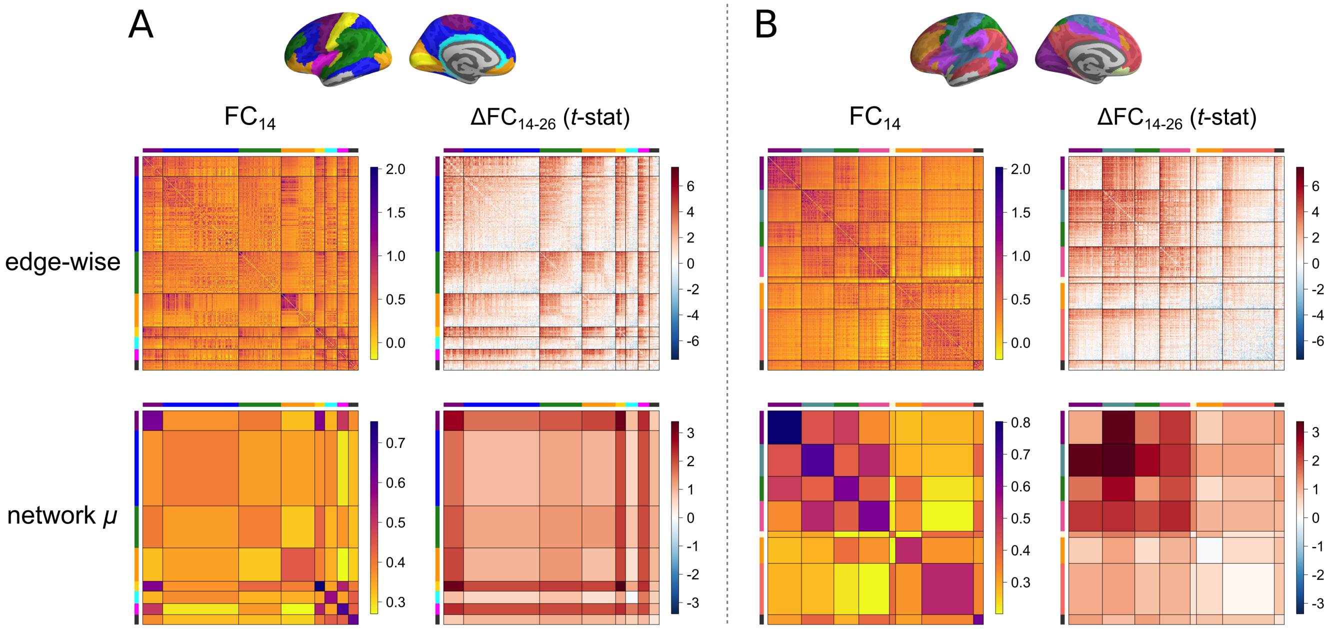

Fig. S6. Maturation of functional connectivity at the edge level. Parameters of linear models extracted from mixed-effect models fitted to FC of individual edges. FC_{14} and ΔFC_{14-26} within and between A) cytoarchitectonic classes of the von Economo atlas (26), and B) resting state networks derived from prior resting state FC analysis by Yeo (20). In both cases, subcortical regions were considered as an additional eighth class/subnetwork (grey). Values are depicted at the level of individual edges ("edge-wise", top row) and as averages of within- and between-network blocks ("network μ", bottom row). In "edge-wise" plots, regions are sorted in decreasing order of average regional value within each network.

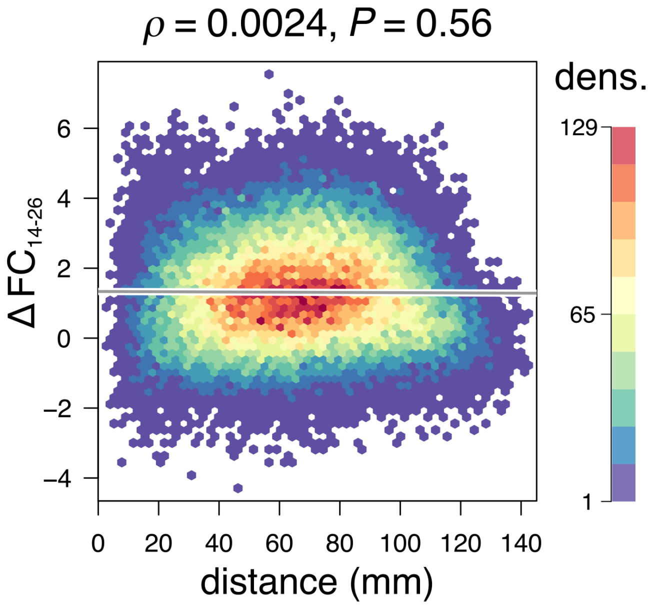

Fig. S7. Relationship of changes in FC to Euclidean distance. Distances spanned by edges were calculated as the Euclidean distance between coordinates of centroid of the connected pair of regions. Changes in FC (ΔFC_{14-26}) were extracted using mixed-effect linear models at the edge level, across participants. The plot is a 2D-histogram of the underlying dense data, with hexagonal bins color-coded by the number of edges located within.

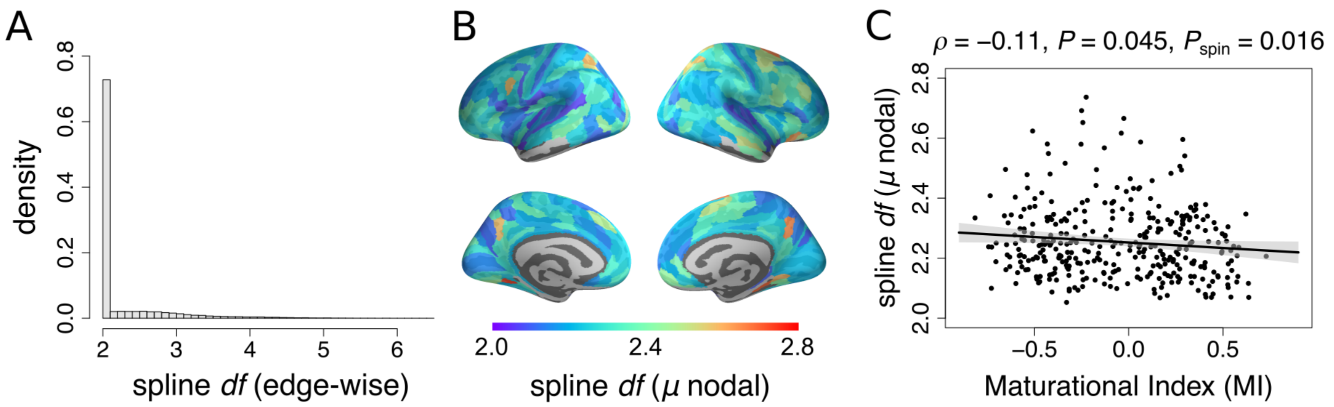

Fig. S8. Inspecting edge-wise trajectories for potential presence of non-linearities. A) Distribution of effective *df* of smoothing splines across edges. Most trajectories are linear: 71.7% trajectories had $df < 2.1$. B) Cortical distribution of average nodal *df* (averaged across all of a node's edges). C) Relationship of the maturational index (MI) with nodal *df*.

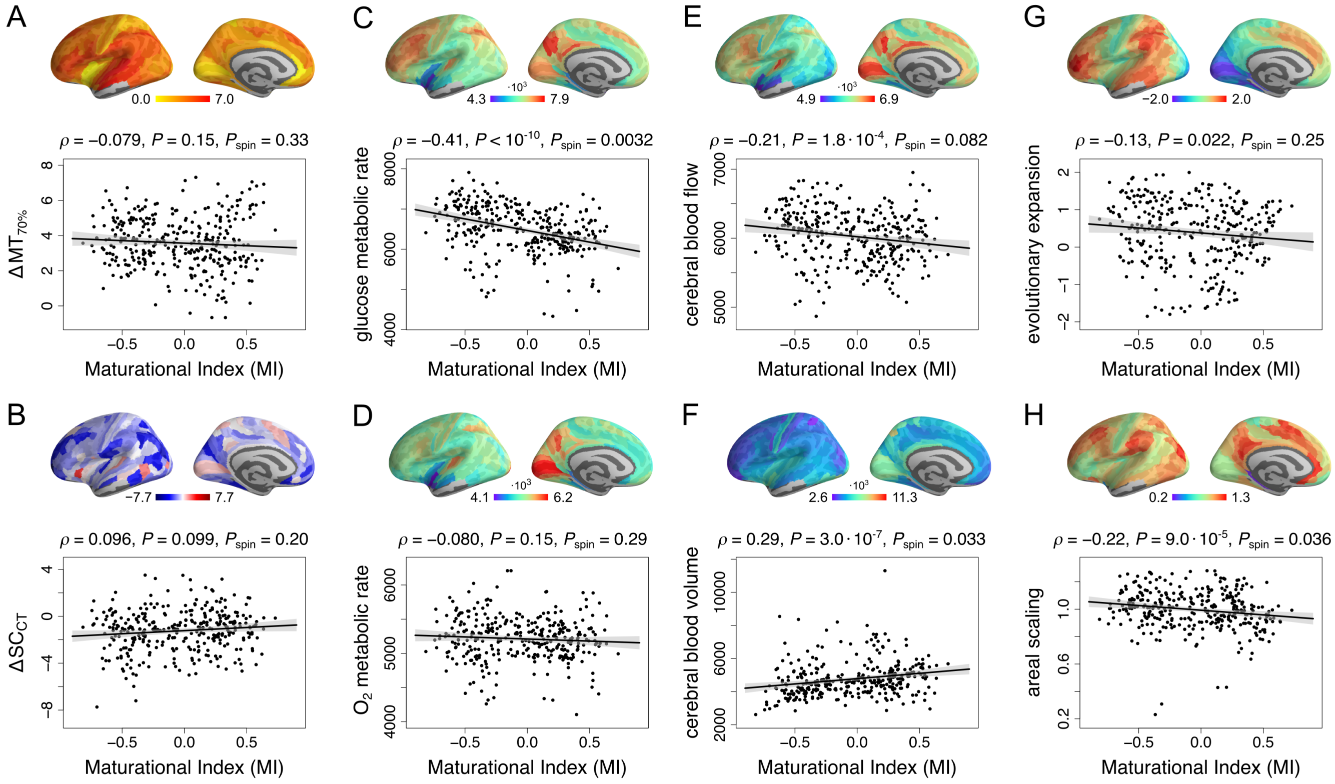

Fig. S9. Relationships between maturational index and additional cortical maps. Spatial correlations between regional maturational index, and eight cortical maps: A) The rate of change of magnetization transfer (MT; a marker of myelination), extracted at 70% depth into cortex as in (3); B) The rate of change of nodal structural covariation of cortical thickness (CT) across participants as in (4); C) metabolic rate of glucose; D) metabolic rate of oxygen; E) cerebral blood flow and F) cerebral blood volume, all (D-F) from (30); G) rate of evolutionary expansion of the cortical surface, from (33); and H) areal scaling of the cortical surface, from (32). All *P*-values reported above were corrected for a total of 12 multiple comparisons (including the four maps in main Fig. 4 and the eight maps above) using the false discovery rate (FDR). For further details see Table S4.

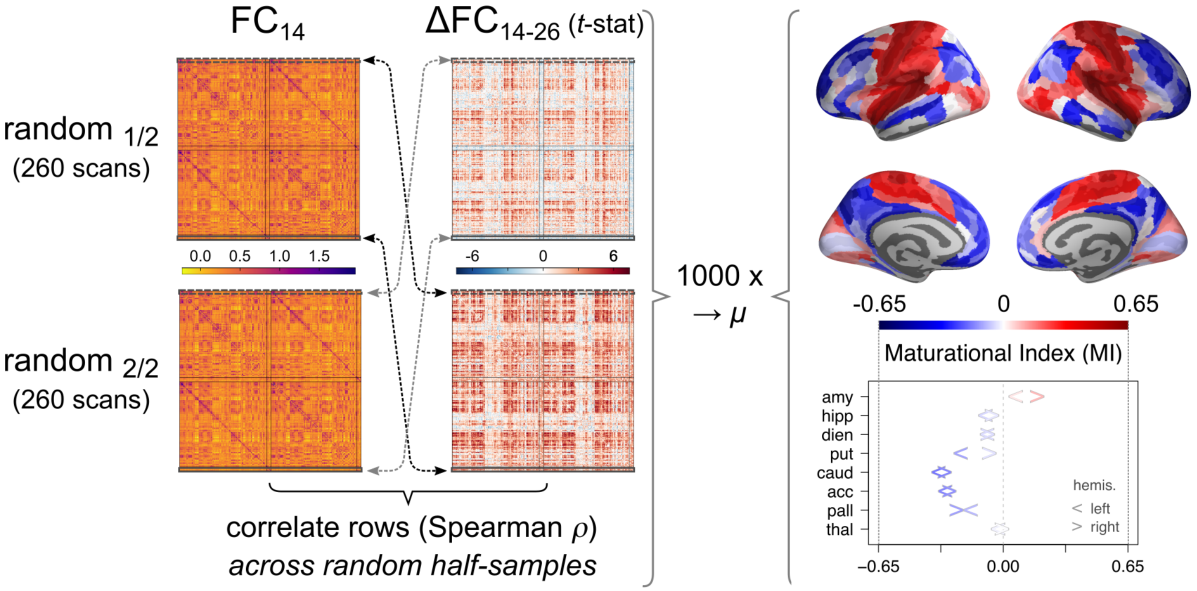

Fig. S10. Association cortical and subcortical 'disruptive' maturation is robust to random half-splitting of the data. The set of 520 scans was randomly split in half, and edge-wise mixed-effect linear models were fitted separately to each half (of 260 scans), leading to two independent estimates of (i) FC at age 14 (FC_{14}), and (ii) the adolescent rate of change of FC (ΔFC_{14-26}). The correlation between the two matrices was then calculated *across* the random half-samples, and averaged between the two split combinations. The results correspond to an average across 1000 random half-splits.

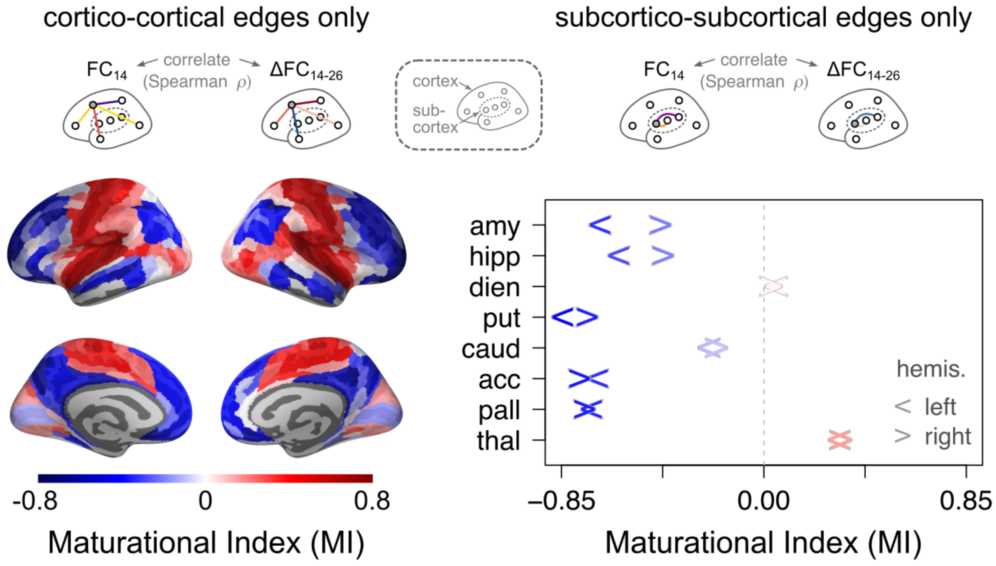

Fig. S11. Association cortical and subcortical 'disruptive' maturation is robust to separate estimation using cortico-cortical and subcortico-subcortical edges. To account for potential differences in temporal signal-to-noise ratio (tSNR) between cortical and subcortical regions, the maturational index was re-estimated by separately using only cortico-cortical edges (left) and subcortico-subcortical edges (right).

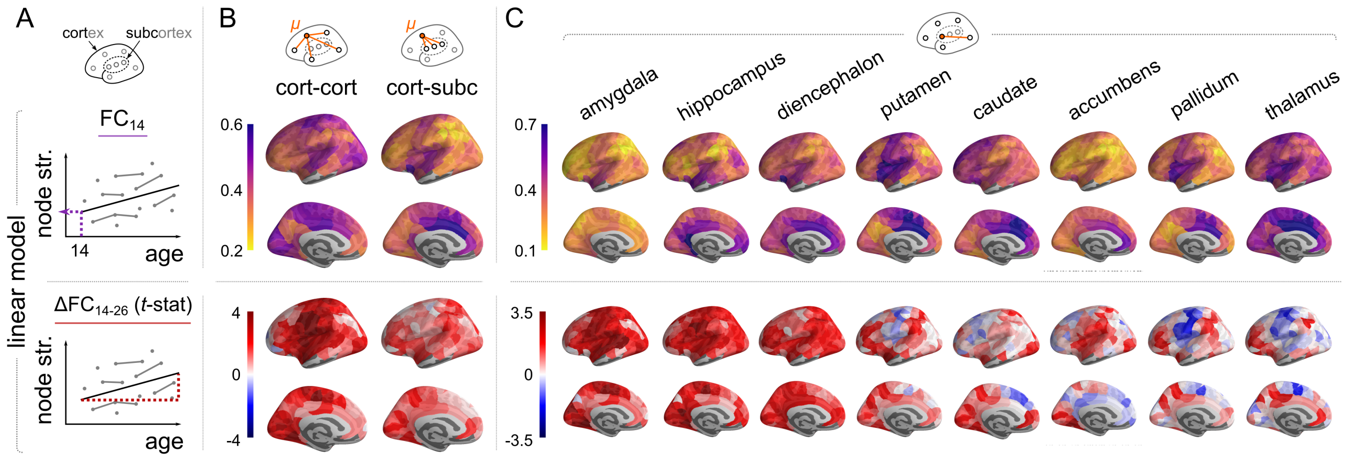

Fig. S12. Replication of main text Fig. 1 in the DK-sub parcellation: Regional strength of functional connectivity of cortical areas and subcortical nuclei at 14 years (FC_{14}) and regional change in strength of connectivity during adolescence (ΔFC_{14-26}). A) Regional strength for each of 282 cortical and 16 subcortical nodes was regressed on a linear function of age for all participants (N = 520 scans from 298 participants; mixed effects model). B) Average cortico-cortical and cortico-subcortical FC_{14} and ΔFC_{14-26}. C) Heterogeneous FC_{14} and ΔFC_{14-26} of individual subcortical nuclei to cortex (subcortical regions are in the same order as main Fig. 1). Due to bilateral symmetry, only left hemispheres are visualised.

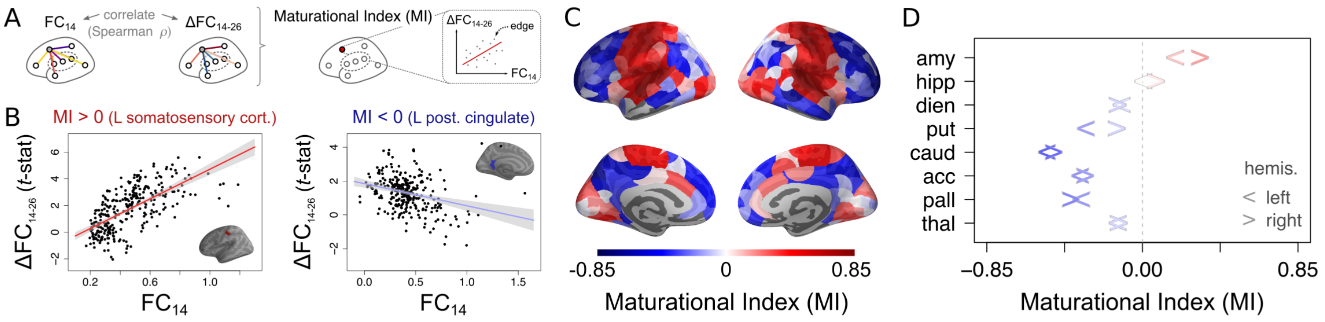

Fig. S13. Replication of main text Fig. 2 in the DK-sub parcellation: Maturational index. A) The maturational index (MI) for each brain region is defined as the correlation of edge-wise baseline FC_{14} versus rate of change ΔFC_{14-26}. Panel B) illustrates this for two example regions, including a positive MI in left somatosensory cortex, and a negative MI in left posterior cingulate cortex. C) Visualisation of the Maturational Index for all cortical regions, and D) subcortical regions (the left/right arrow corresponds to left/right hemisphere).

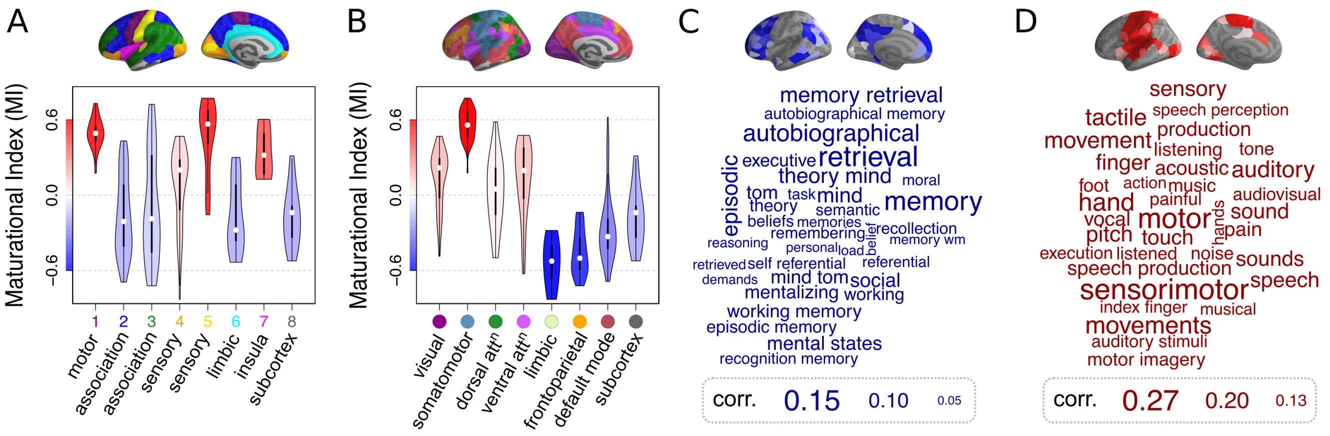

Fig. S14. Replication of main text Fig. 3 in the DK-sub parcellation: Maturational index in anatomical and psychological context. A) Distribution of maturational index for each cytoarchitectonic class of the von Economo atlas (26), and B) for resting state networks derived from prior resting state FC analysis by Yeo (20). In both cases, subcortical regions were considered as an additional eighth class/subnetwork. The violin plots are coloured by average MI within the corresponding class of regions. C-D) Word clouds of cognitive terms associated with cortical brain regions that have C) disruptive (blue) or D) conservative (red) modes of development (Neurosynth decoding (28)). The size of cognitive terms corresponds to the correlation of corresponding meta-analytic maps generated by Neurosynth with each of the two modes (top).

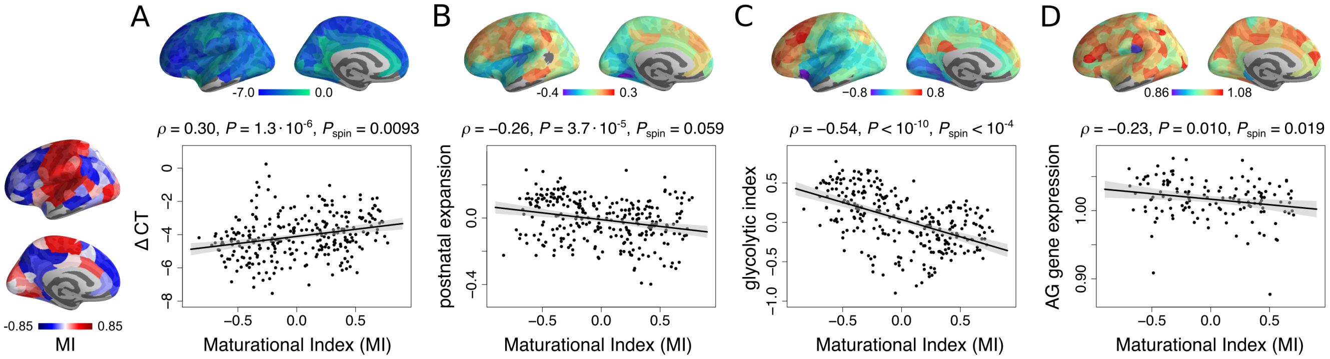

Fig. S15. Replication of main text Fig. 4 in the DK-sub parcellation: Disruptive and conservative modes of fMRI maturation in developmental and metabolic context. A) Maturational index was positively correlated with ΔCT - regions which had disruptive development (MI<0) had faster rates of cortical thickness (CT) shrinkage during adolescence. B) MI was negatively correlated with a prior map of postnatal cortical surface area - disruptive maturation was greater in regions that showed greatest expansion after birth. C) MI was negatively correlated with a prior map of the glycolytic index, a measure of aerobic glycolysis (AG); and D) MI was negatively correlated with a prior map of brain regional expression of AG-related genes. (33).

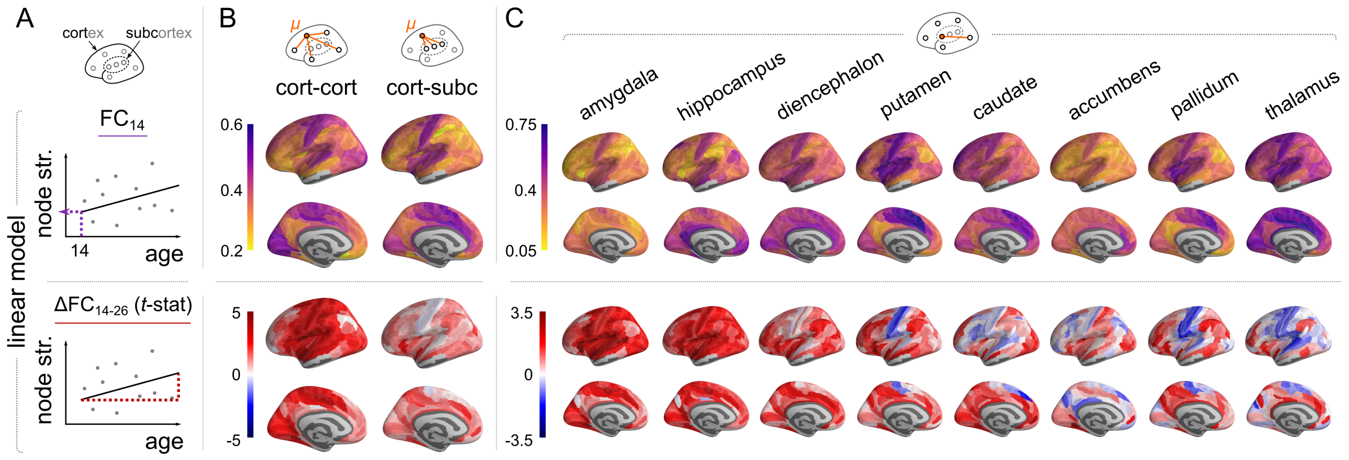

Fig. S16. Replication of main text Fig. 1 in cross-sectional data: Regional strength of functional connectivity of cortical areas and subcortical nuclei at 14 years (FC_{14}) and regional change in strength of connectivity during adolescence (ΔFC_{14-26}). A) Regional strength for each of 330 cortical and 16 subcortical nodes was regressed on a linear function of age for all participants (N = 298 scans from 298 participants; fixed effects model). B) Average cortico-cortical and cortico-subcortical FC_{14} and ΔFC_{14-26}. C) Heterogeneous FC_{14} and ΔFC_{14-26} of individual subcortical nuclei to cortex (subcortical regions are in the same order as main Fig. 1). Due to bilateral symmetry, only left hemispheres are visualised.

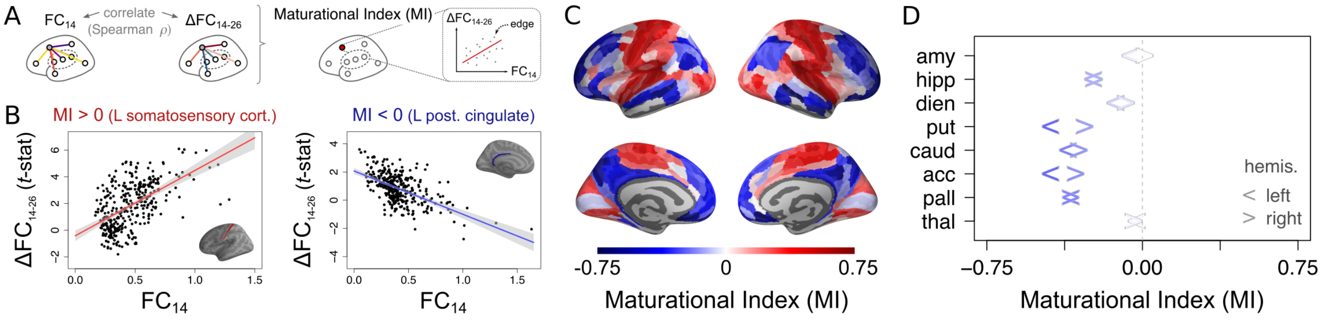

Fig. S17. Replication of main text Fig. 2 in cross-sectional data: Maturational index. A) The maturational index (MI) for each brain region is defined as the correlation of edge-wise baseline FC_{14} versus rate of change ΔFC_{14-26}. Panel B) illustrates this for two example regions, including a positive MI in left somatosensory cortex, and a negative MI in left posterior cingulate cortex. C) Visualisation of the Maturational Index for all cortical regions, and D) subcortical regions (the left/right arrow corresponds to left/right hemisphere).

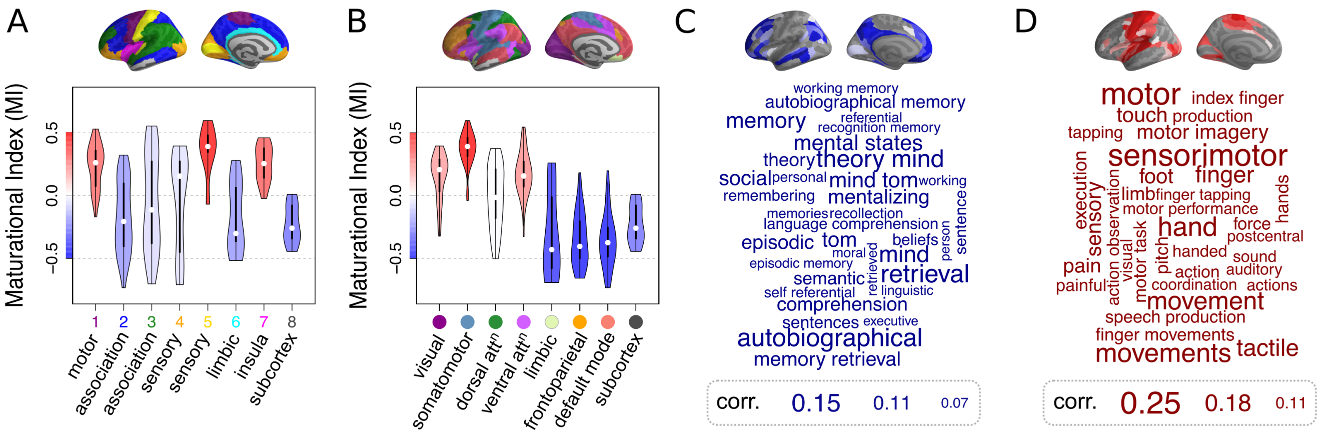

Fig. S18. Replication of main text Fig. 3 in cross-sectional data: Maturational index in anatomical and psychological context. A) Distribution of maturational index for each cytoarchitectonic class of the von Economo atlas (26), and B) for resting state networks derived from prior resting state FC analysis by Yeo (20). In both cases, subcortical regions were considered as an additional eighth class/subnetwork. The violin plots are coloured by average MI within the corresponding class of regions. C-D) Word clouds of cognitive terms associated with cortical brain regions that have C) disruptive (blue) or D) conservative (red) modes of development (Neurosynth decoding (28)). The size of cognitive terms corresponds to the correlation of corresponding meta-analytic maps generated by Neurosynth with each of the two modes (top).

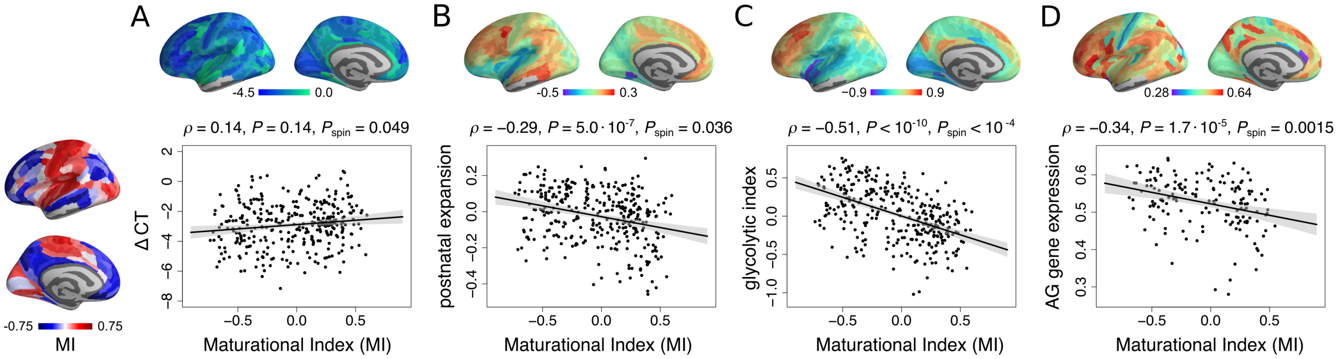

Fig. S19. Replication of main text Fig. 4 in cross-sectional data: Disruptive and conservative modes of fMRI maturation in developmental and metabolic context. A) Maturational index was positively correlated with ΔCT - regions which had disruptive development (MI<0) had faster rates of cortical thickness (CT) shrinkage during adolescence. B) MI was negatively correlated with a prior map of postnatal cortical surface area - disruptive maturation was greater in regions that showed greatest expansion after birth. C) MI was negatively correlated with a prior map of the glycolytic index, a measure of aerobic glycolysis (AG); and D) MI was negatively correlated with a prior map of brain regional expression of AG-related genes. (33).

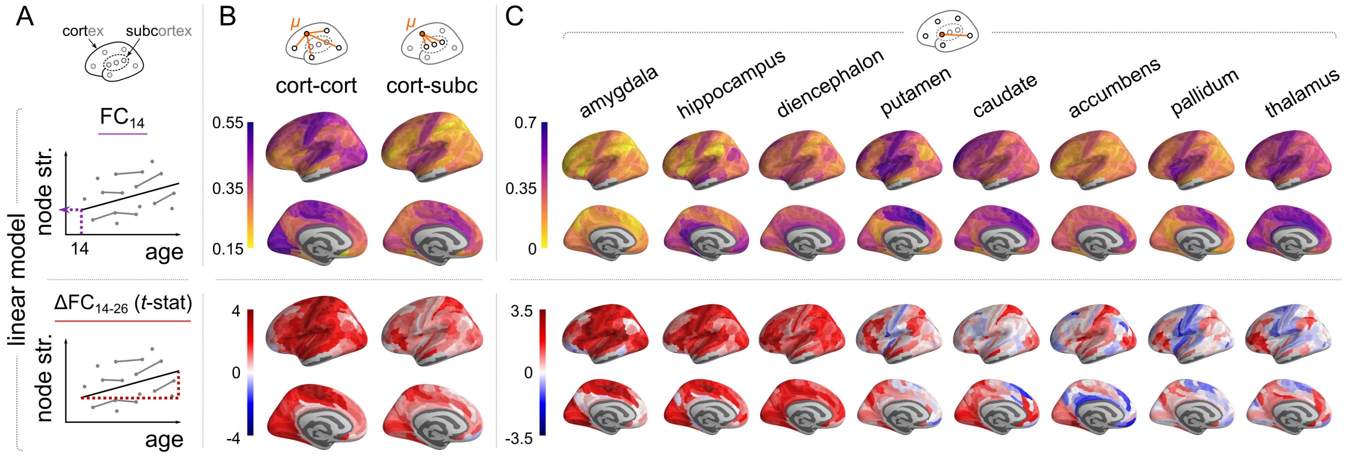

Fig. S20. Replication of main text Fig. 1 in data from a single scanner: Regional strength of functional connectivity of cortical areas and subcortical nuclei at 14 years (FC_{14}) and regional change in strength of connectivity during adolescence (ΔFC_{14-26}). A) Regional strength for each of 330 cortical and 16 subcortical nodes was regressed on a linear function of age for all participants (N = 396 scans from 236 participants; mixed effects model). B) Average cortico-cortical and cortico-subcortical FC_{14} and ΔFC_{14-26}. C) Heterogeneous FC_{14} and ΔFC_{14-26} of individual subcortical nuclei to cortex (subcortical regions are in the same order as main Fig. 1). Due to bilateral symmetry, only left hemispheres are visualised.

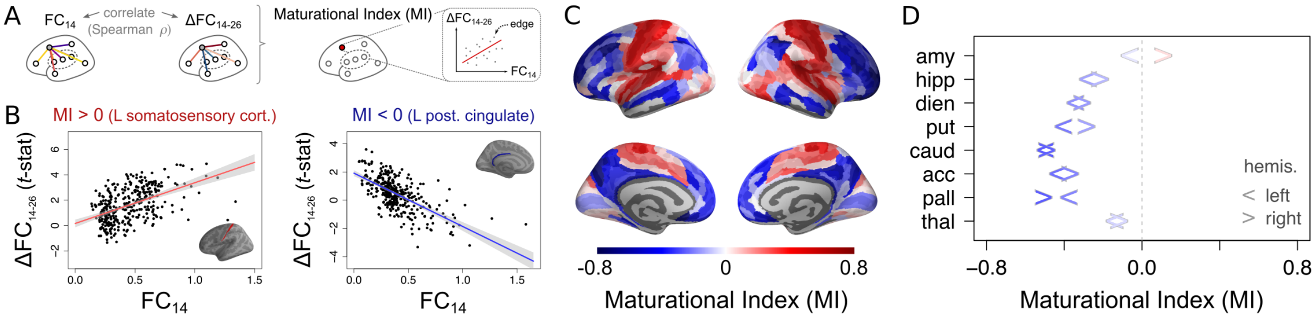

Fig. S21. Replication of main text Fig. 2 in data from a single scanner: Maturational index. A) The maturational index (MI) for each brain region is defined as the correlation of edge-wise baseline FC_{14} versus rate of change ΔFC_{14-26}. Panel B) illustrates this for two example regions, including a positive MI in left somatosensory cortex, and a negative MI in left posterior cingulate cortex. C) Visualisation of the Maturational Index for all cortical regions, and D) subcortical regions (the left/right arrow corresponds to left/right hemisphere).

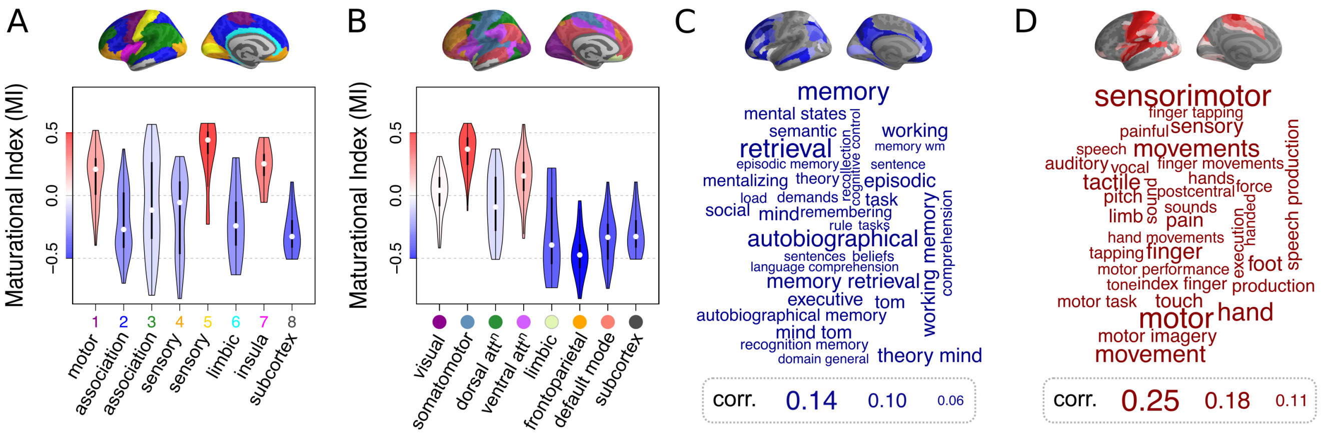

Fig. S22. Replication of main text Fig. 3 in data from a single scanner: Maturational index in anatomical and psychological context. A) Distribution of maturational index for each cytoarchitectonic class of the von Economo atlas (26), and B) for resting state networks derived from prior resting state FC analysis by Yeo (20). In both cases, subcortical regions were considered as an additional eighth class/subnetwork. The violin plots are coloured by average MI within the corresponding class of regions. C-D) Word clouds of cognitive terms associated with cortical brain regions that have C) disruptive (blue) or D) conservative (red) modes of development (Neurosynth decoding (28)). The size of cognitive terms corresponds to the correlation of corresponding meta-analytic maps generated by Neurosynth with each of the two modes (top).

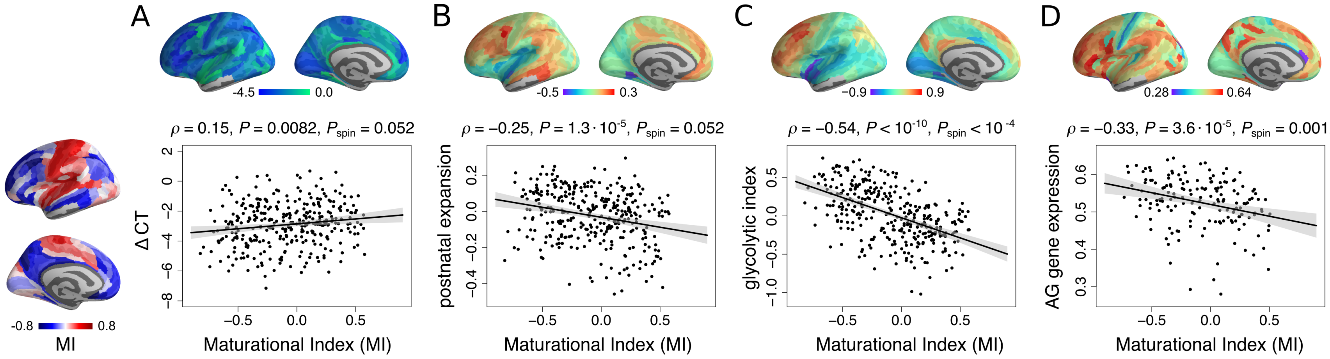

Fig. S23. Replication of main text Fig. 4 in data from a single scanner: Disruptive and conservative modes of fMRI maturation in developmental and metabolic context. A) Maturational index was positively correlated with ΔCT - regions which had disruptive development (MI<0) had faster rates of cortical thickness (CT) shrinkage during adolescence. B) MI was negatively correlated with a prior map of postnatal cortical surface area - disruptive maturation was greater in regions that showed greatest expansion after birth. C) MI was negatively correlated with a prior map of the glycolytic index, a measure of aerobic glycolysis (AG); and D) MI was negatively correlated with a prior map of brain regional expression of AG-related genes. (33).

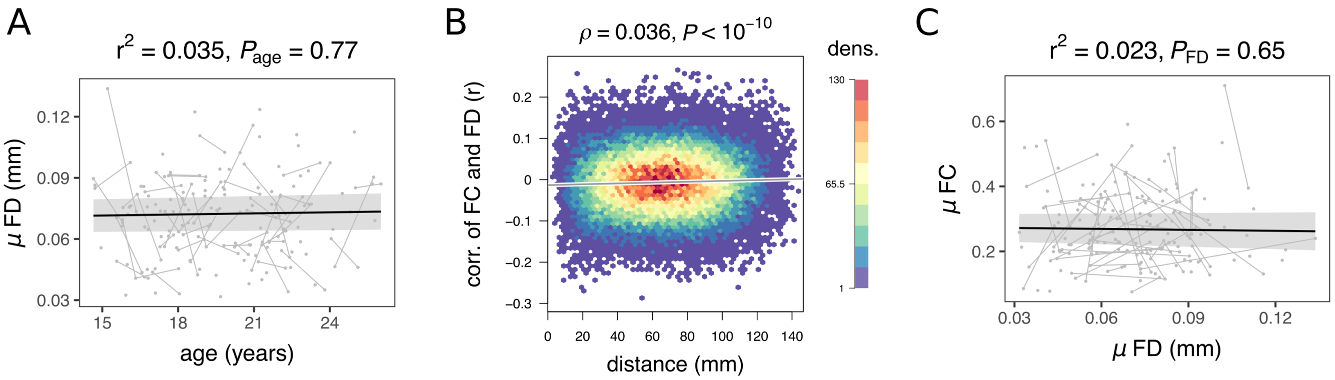

Fig. S24. Effects of head motion on functional connectivity, in a low-motion subset of data. A) Average participant motion (quantified as mean FD) does not change with age (the pseudo-r^2 for generalized mixed-effect models (GLMMs) includes variance in mean FD explained by age, as well as sex and scanner site). B) The correlation between FC at each edge and the corresponding participant's motion (across participants) does not show a strong relationship with the Euclidean distance spanned by edges. The average edge-wise correlation between FC and motion (average of data along the y-axis) is near-zero. C) Mean participant motion is not related to mean FC across participants (the pseudo-r^2 for GLMMs includes variance in mean FC explained by mean FD, as well as sex). Panel B depicts a 2D-histogram of the underlying dense data, with hexagonal bins color-coded by the number of edges located within.

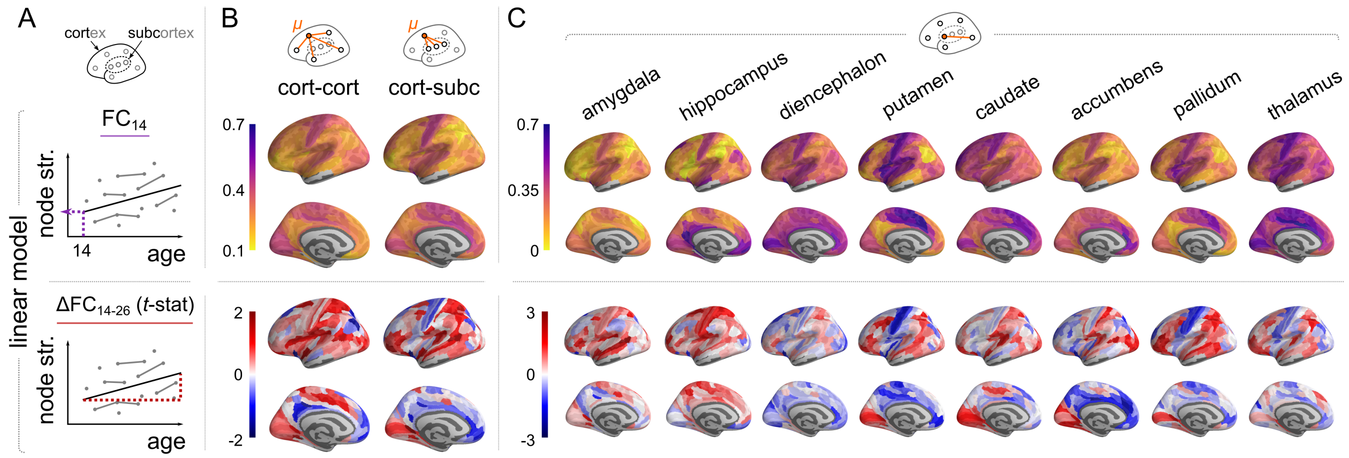

Fig. S25. Replication of main text Fig. 1 in a low-motion subset of data: Regional strength of functional connectivity of cortical areas and subcortical nuclei at 14 years (FC_{14}) and regional change in strength of connectivity during adolescence (ΔFC_{14-26}). A) Regional strength for each of 330 cortical and 16 subcortical nodes was regressed on a linear function of age for all participants (N = 182 scans from 132 participants; mixed effects model). B) Average cortico-cortical and cortico-subcortical FC_{14} and ΔFC_{14-26}. C) Heterogeneous FC_{14} and ΔFC_{14-26} of individual subcortical nuclei to cortex (subcortical regions are in the same order as main Fig. 1). Due to bilateral symmetry, only left hemispheres are visualised.

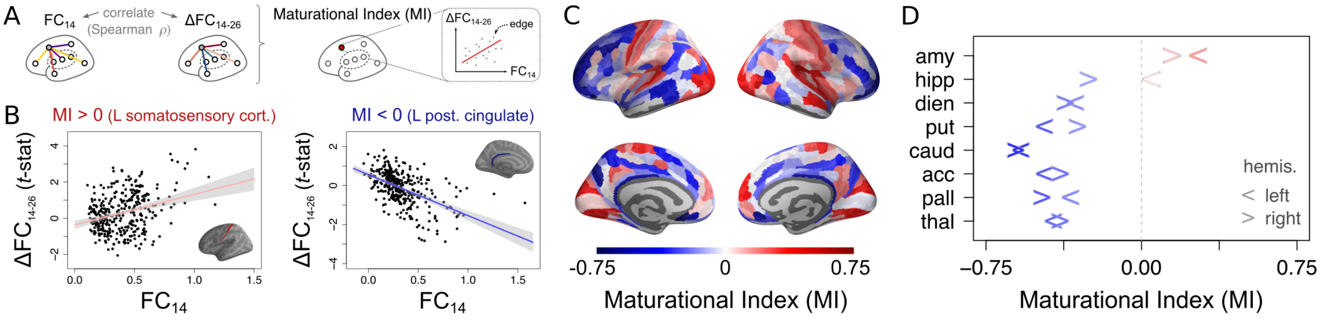

Fig. S26. Replication of main text Fig. 2 in a low-motion subset of data: Maturational index. A) The maturational index (MI) for each brain region is defined as the correlation of edge-wise baseline FC_{14} versus rate of change ΔFC_{14-26}. Panel B) illustrates this for two example regions, including a positive MI in left somatosensory cortex, and a negative MI in left posterior cingulate cortex. C) Visualisation of the Maturational Index for all cortical regions, and D) subcortical regions (the left/right arrow corresponds to left/right hemisphere).

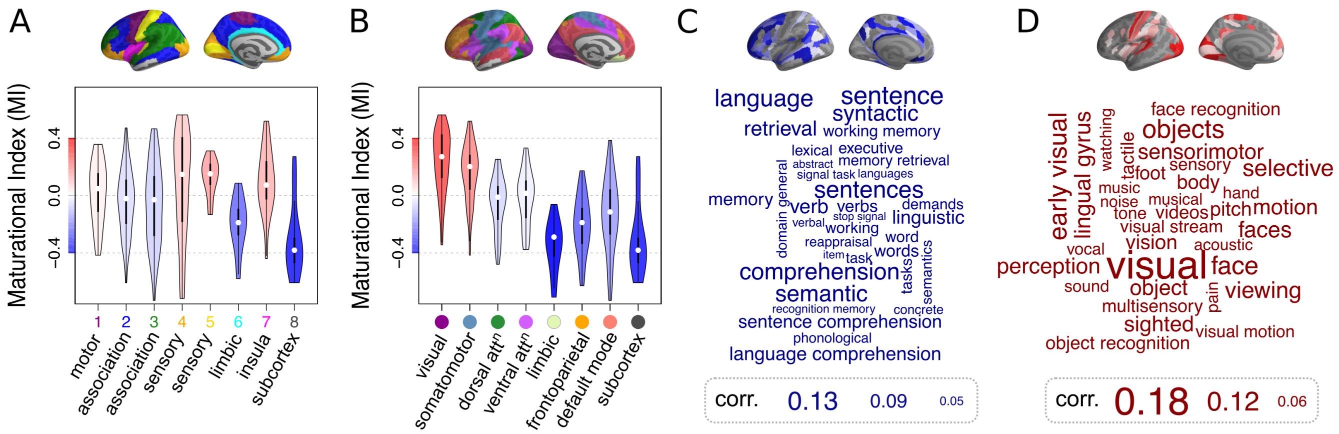

Fig. S27. Replication of main text Fig. 3 in a low-motion subset of data: Maturational index in anatomical and psychological context. A) Distribution of maturational index for each cytoarchitectonic class of the von Economo atlas (26), and B) for resting state networks derived from prior resting state FC analysis by Yeo (20). In both cases, subcortical regions were considered as an additional eighth class/subnetwork. The violin plots are coloured by average MI within the corresponding class of regions. C-D) Word clouds of cognitive terms associated with cortical brain regions that have C) disruptive (blue) or D) conservative (red) modes of development (Neurosynth decoding (28)). The size of cognitive terms corresponds to the correlation of corresponding meta-analytic maps generated by Neurosynth with each of the two modes (top).

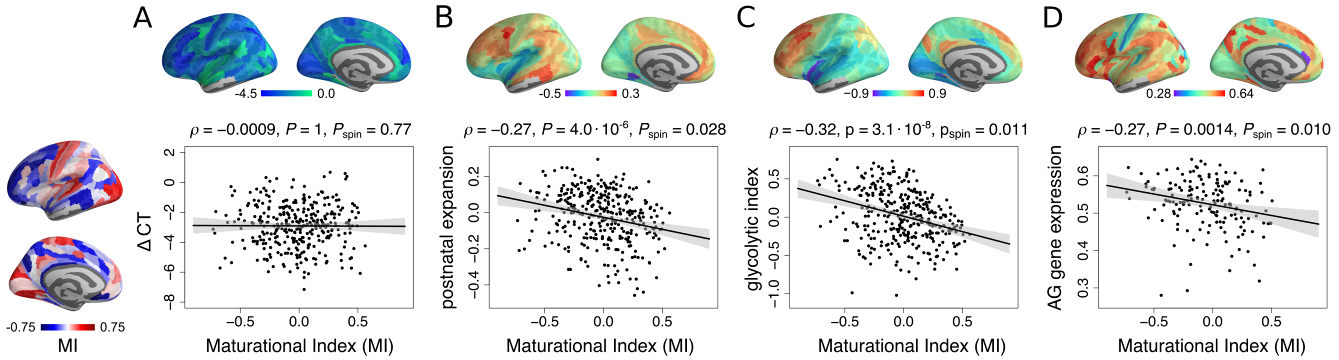

Fig. S28. Replication of main text Fig. 4 in a low-motion subset of data: Disruptive and conservative modes of fMRI maturation in developmental and metabolic context. A) Maturational index was not correlated with ΔCT in the low-motion subset of data. B) MI was negatively correlated with a prior map of postnatal cortical surface area - disruptive maturation was greater in regions that showed greatest expansion after birth. C) MI was negatively correlated with a prior map of the glycolytic index, a measure of aerobic glycolysis (AG); and D) MI was negatively correlated with a prior map of brain regional expression of AG-related genes. (33).

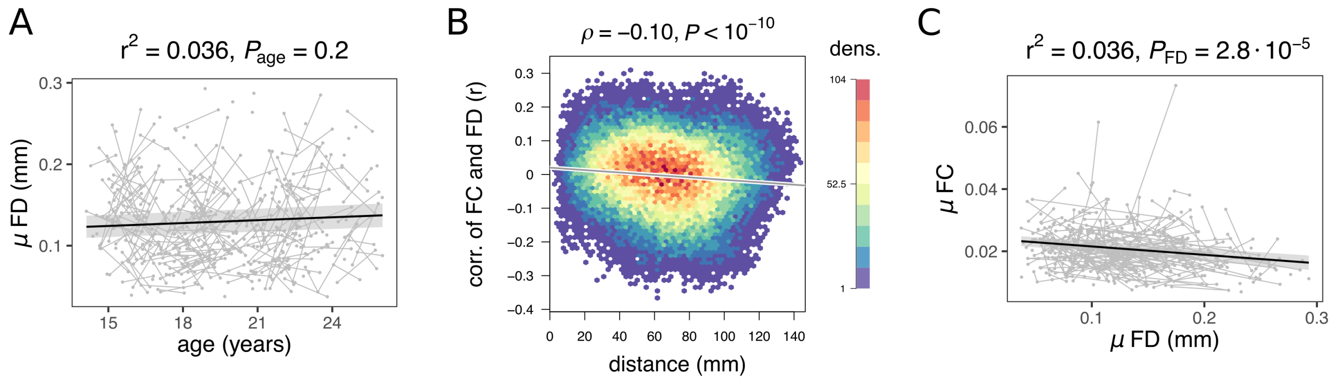

Fig. S29. Effects of head motion on functional connectivity, in data processed with global signal regression (GSR). A) Average participant motion (quantified as mean FD) does not change with age (the pseudo-r^2 for generalized mixed-effect models (GLMMs) includes variance in mean FD explained by age, as well as sex and scanner site). B) The correlation between FC at each edge and the corresponding participant's motion (across participants) does not show a strong relationship with the Euclidean distance spanned by edges. The average edge-wise correlation between FC and motion (average of data along the y-axis) is near-zero. C) Mean participant motion is weakly related to mean FC across participants (the pseudo-r^2 for GLMMs includes variance in mean FC explained by mean FD, as well as sex). Panel B depicts a 2D-histogram of the underlying dense data, with hexagonal bins color-coded by the number of edges located within.

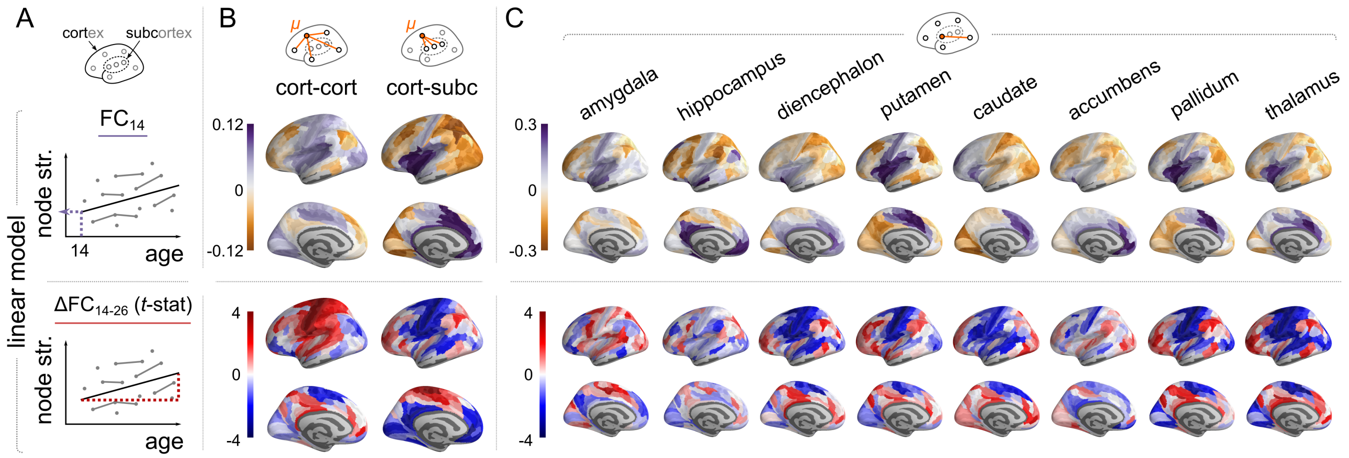

Fig. S30. Replication of main text Fig. 1 in data processed with global signal regression (GSR): Regional strength of functional connectivity of cortical areas and subcortical nuclei at 14 years (FC_{14}) and regional change in strength of connectivity during adolescence (ΔFC_{14-26}). A) Regional strength for each of 330 cortical and 16 subcortical nodes was regressed on a linear function of age for all participants (N = 520 scans from 298 participants; mixed effects model). B) Average cortico-cortical and cortico-subcortical FC_{14} and ΔFC_{14-26}. C) Heterogeneous FC_{14} and ΔFC_{14-26} of individual subcortical nuclei to cortex (subcortical regions are in the same order as main Fig. 1). Due to bilateral symmetry, only left hemispheres are visualised.

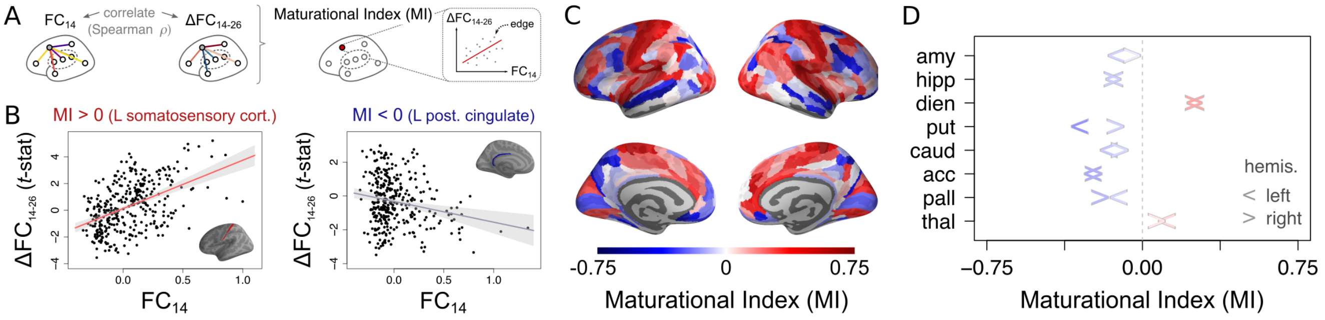

Fig. S31. Replication of main text Fig. 2 in data processed with global signal regression (GSR): Maturational index. A) The maturational index (MI) for each brain region is defined as the correlation of edge-wise baseline FC_{14} versus rate of change ΔFC_{14-26}. Panel B) illustrates this for two example regions, including a positive MI in left somatosensory cortex, and a negative MI in left posterior cingulate cortex. C) Visualisation of the Maturational Index for all cortical regions, and D) subcortical regions (the left/right arrow corresponds to left/right hemisphere).

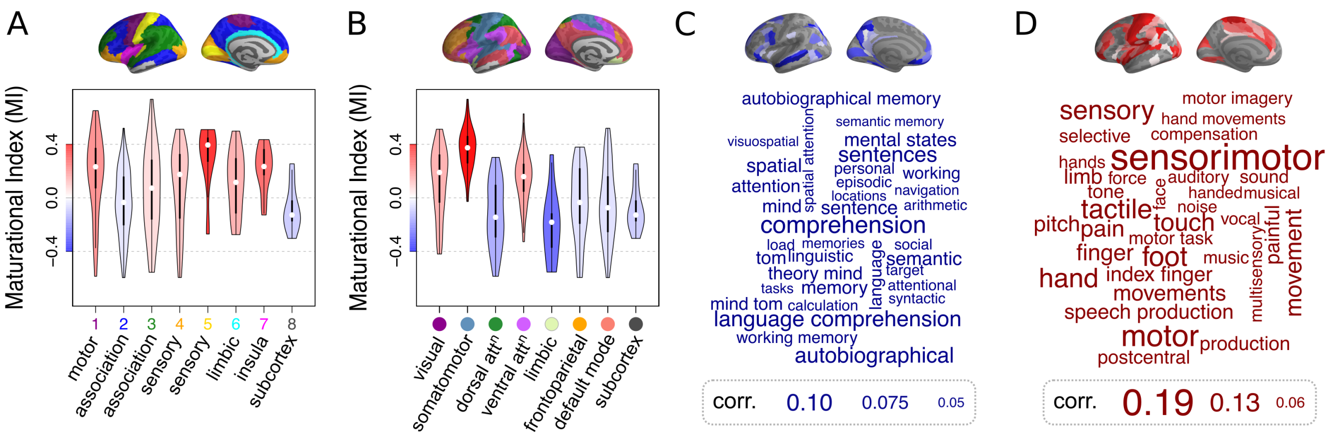

Fig. S32. Replication of main text Fig. 3 in data processed with global signal regression (GSR): Maturational index in anatomical and psychological context. A) Distribution of maturational index for each cytoarchitectonic class of the von Economo atlas (26), and B) for resting state networks derived from prior resting state FC analysis by Yeo (20). In both cases, subcortical regions were considered as an additional eighth class/subnetwork. The violin plots are coloured by average MI within the corresponding class of regions. C-D) Word clouds of cognitive terms associated with cortical brain regions that have C) disruptive (blue) or D) conservative (red) modes of development (Neurosynth decoding (28)). The size of cognitive terms corresponds to the correlation of corresponding meta-analytic maps generated by Neurosynth with each of the two modes (top).

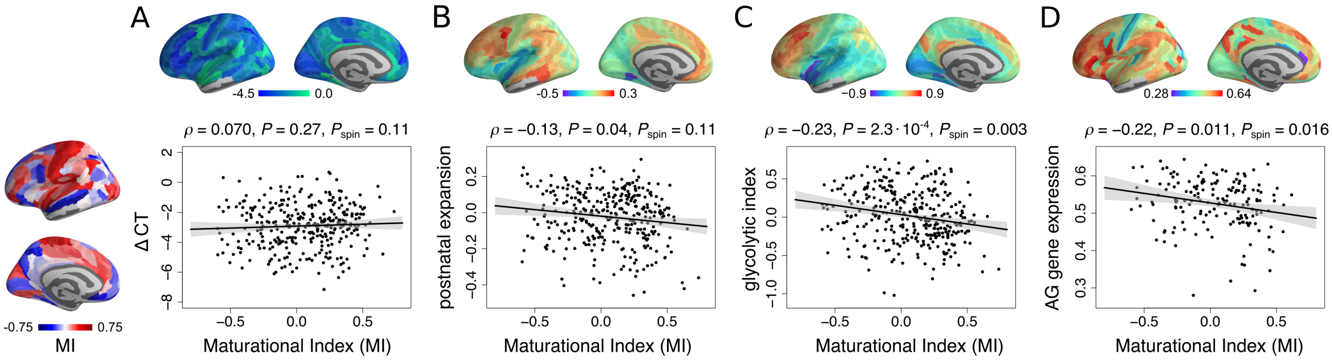

Fig. S33. Replication of main text Fig. 4 in data processed with global signal regression (GSR): Disruptive and conservative modes of fMRI maturation in developmental and metabolic context. A) Maturational index was not correlated with ΔCT in data processed with GSR. B) MI was negatively correlated with a prior map of postnatal cortical surface area - disruptive maturation was greater in regions that showed greatest expansion after birth. C) MI was negatively correlated with a prior map of the glycolytic index, a measure of aerobic glycolysis (AG); and D) MI was negatively correlated with a prior map of brain regional expression of AG-related genes. (33).

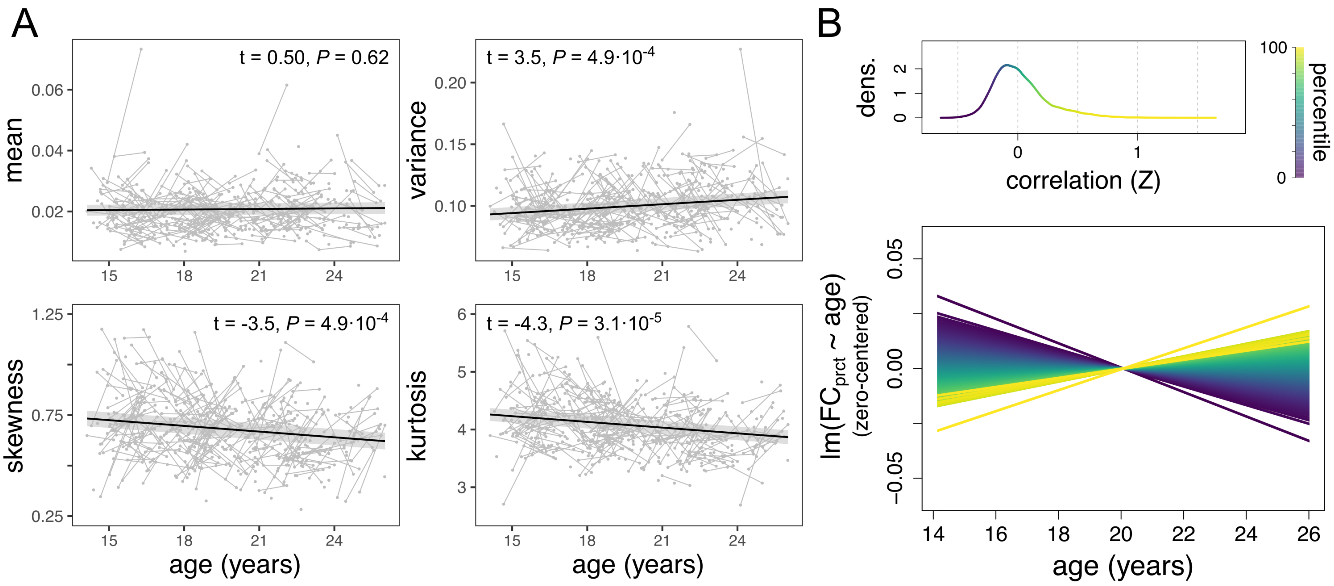

Fig. S34. Replication of SI Fig. S3 in data processed with global signal regression (GSR): Global trajectories of the correlation distribution. A) Trajectories of the four moments of the correlation distribution as a function of age: (i) mean, (ii) variance, (iii) skewness and (iv) kurtosis. B) Example distribution, colour-coded by percentile. Higher percentiles of the correlation distribution strengthen faster, as indicated by increasing slopes of linear models fit to values of correlation at increasing percentiles. This is visualised using a plot of all the linear models as a function of age (centered along the y-axis for visual clarity).

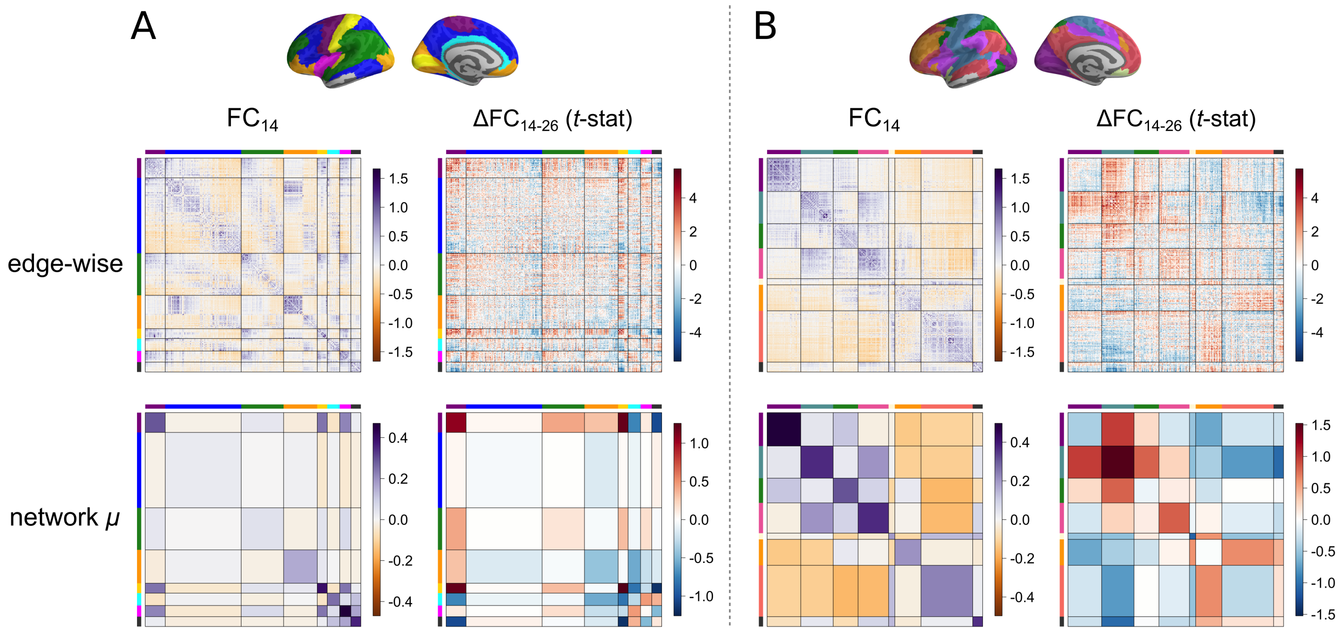

Fig. S35. Replication of SI Fig. S6 in data processed with global signal regression (GSR): Maturation of functional connectivity at the edge level. Parameters of linear models extracted from mixed-effect models fitted to FC of individual edges. FC_{14} and ΔFC_{14-26} within and between A) cytoarchitectonic classes of the von Economo atlas (26), and B) resting state networks derived from prior resting state FC analysis by Yeo (20). In both cases, subcortical regions were considered as an additional eighth class/subnetwork (grey). Values are depicted at the level of individual edges ("edge-wise", top row) and as averages of within- and between-network blocks ("network μ", bottom row). In "edge-wise" plots, regions are sorted in decreasing order of average regional value within each network.

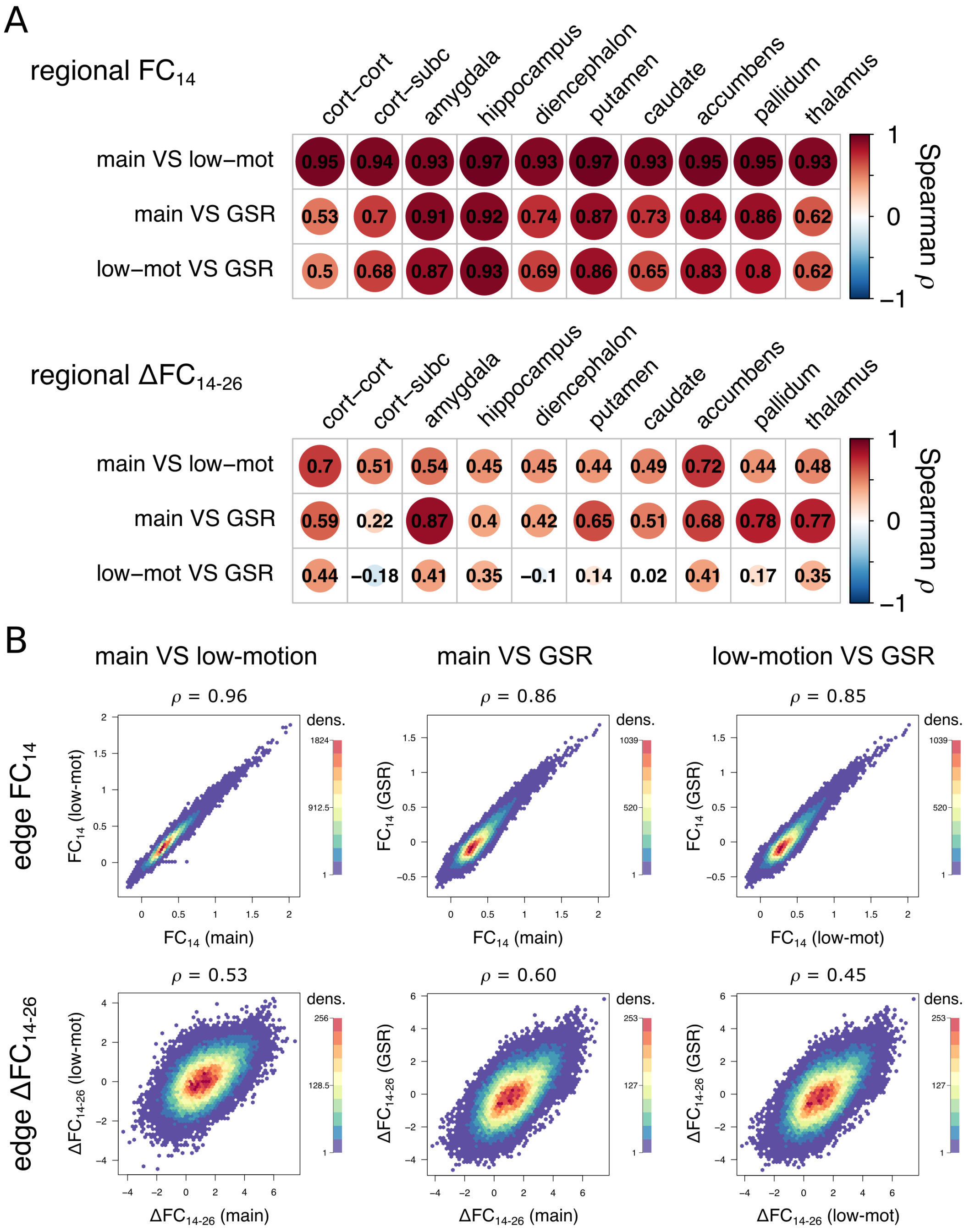

Fig. S36. Comparison of regional and edge FC_{14} and ΔFC_{14-26} across denoising pipelines. A) Pairwise comparison of regional developmental parameters obtained from three different denoising pipelines: FC_{14} (top) and ΔFC_{14-26} (bottom). The cortical maps being compared are presented in main Fig. 1 and analogous supplementary figures, Fig. S25 and Fig. S30. B) Pairwise comparison of edge-wise developmental parameters obtained from three different denoising pipelines: FC_{14} (top) and ΔFC_{14-26} (bottom). Panels depict 2D-histograms of the underlying dense data, with hexagonal bins color-coded by the number of edges located within.

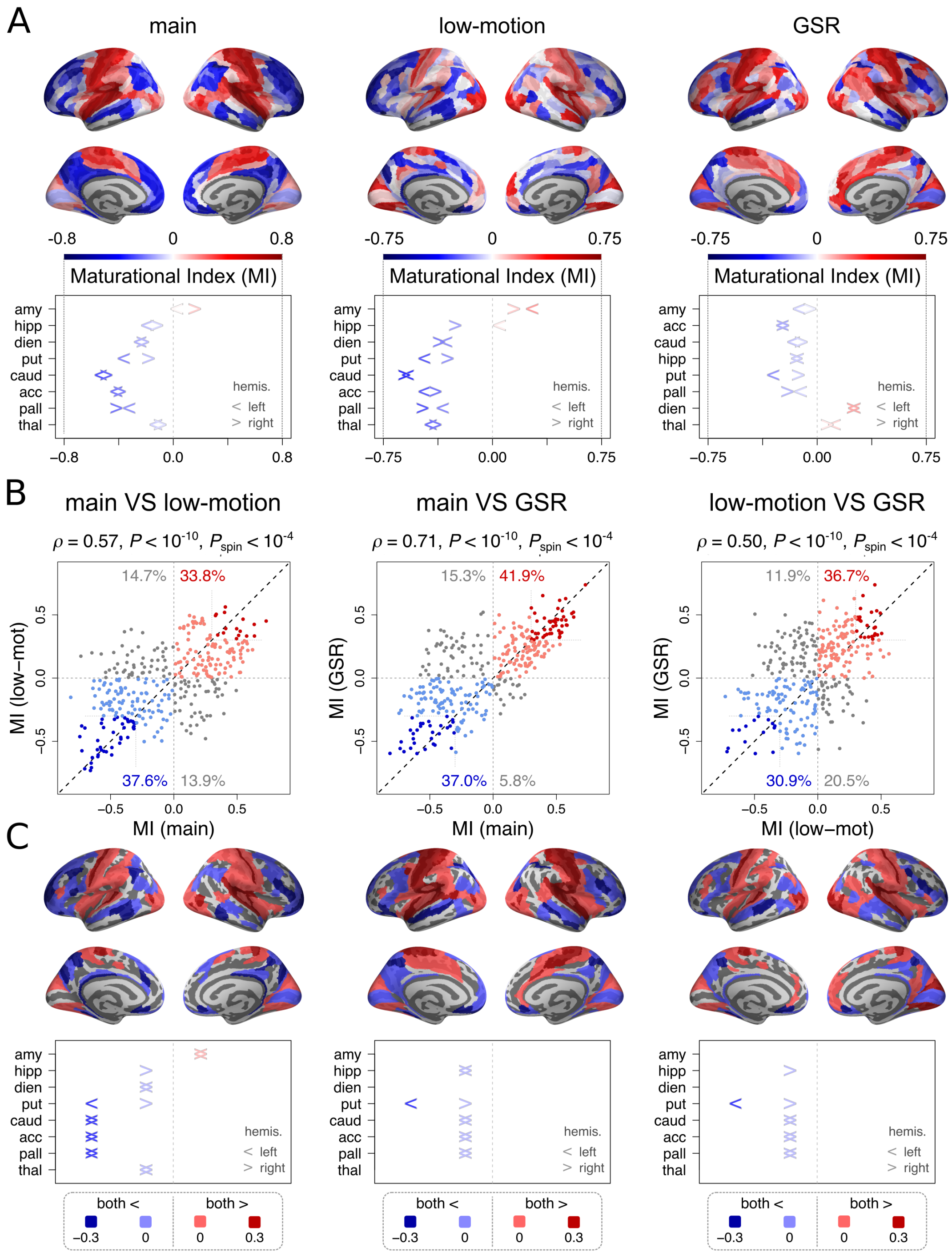

Fig. S37. Comparison of Maturational Index across denoising pipelines. A) Maturational Index obtained from three different denoising pipelines (as presented in main Fig. 2C, Fig. S26C and Fig. S31C). B) Correlations between pairs of MI maps. Quadrants are color-coded based on agreement of MI signs of corresponding regions, and percentages of regions in each quadrant are indicated within. Within agreeing quadrants (i.e. both > 0 or both < 0), regions are further color-coded based based on magnitude of MI: dark markers correspond to both MI > 0.3 (or both MI < -0.3). C) Cortical and subcortical plots of sign overlap, with color-coding for regions with agreeing signs corresponding to panels B, and legends below.

Paper	Age (y)	# Sub.	Motion $\star$	GSR	Nodes	Edges	Thresh.	Results
Fair<br>PNAS<br>2007 (40)	7-31	139	motion match	yes	39	Pears. r	r $\geq$ 0.1	• sub-networks segregate with age<br>• long edges increase in strength, short edges decrease
Fair<br>PLoS CB<br>2009 (41)	7-31	210	none	yes	34	Pears. r	r $\geq$ 0.1	• shift from 'local' to 'distributed' communities<br>• community assignment changes; modularity stable<br>• path length, clustering, small-worldness stable
Supekar<br>PLoS CB<br>2009 (42)	7-22	45	none	no	90	wav. corr.	binary 1-99%	• path length (λ), efficiency, clustering (γ), SWI σ stable<br>• paralimbic weights incr., subcortical weights decr.<br>• long edges increase, short edges decrease
Stevens<br>HBM<br>2009 (43)	12-30	100	none	no	voxels	Grang. caus.	N/A	• linear (or log) decrease in causal interactions,<br>esp. betw. lateral prefrontal-parietal circuits and DMN
Dosenb.<br>Science<br>2010 (44)	7-30	238<br>+195	none	yes	160	Pears. r	none	• strengthening edges longer than weakening edges<br>• within-module edges strengthen, betw.-module weaken<br>• MVPA explains 55% variance of changes with age
Anderson<br>Brain Con.<br>2011 (45)	7-35	1278	none	no	7266	Pears. r	none	• connectivity decrease betw. attention control and DMN<br>• DMN "boundaries sharpen" (conn. within incr.)<br>• slight decr.in connectivity within frontoparietal ROIs
Satterthw.<br>NeuroIm.<br>2013 (46)	8-22	780	motion match	• yes<br>• no<br>(both)	264	Pears. r	none	• motion inflates effects of age & distance-dependence<br>• motion dampens incr. in intra-modular connectivity<br>+ large N needed to detect changes in conn. w/ age
Hwang<br>Cer. Cor.<br>2013 (47)	10-20	99	time despike	no	• voxels<br>• 160	Pears. r	mult.<br>wei.<br>+ bin.	• hub architecture stable<br>• early incr. betw. frontal hubs & cort. + subc. non-hubs<br>• late incr. betw. cerebellar hubs & cortical non-hubs
Wu<br>PLoS One<br>2013 (48)	5.7-18.4	60	none	yes	90	Pears. r	binary 20-35%	• path length (PL, λ), efficiency, clust. (cc) stable<br>• clustering (γ), SWI σ, modularity Q increase<br>• nodal changes in degree, efficiency and betw. cent.
Grayson<br>PLoS One<br>2014 (49)	7-35	21	scrub.	yes	219	Pears. r	binary 1-10%	• rich club strengthening (rich $\rightarrow$ richer & poor $\rightarrow$ rich)<br>• strengthening edges: somatosensory, insula (NBS)<br>+ structural (DWI) rich club organisation stable
Satterthw.<br>PNAS<br>2014 $\ast$ (21)	8-22	922	covaried	N/A	voxels	Pears. r	N/A	• perfusion decr. nonlinear (min. $\sim$17y), esp. assoc. cortex<br>+ trajectory diff. betw. females (decr. U) and males ($\sim$lin.)<br>(sex differences appear only mid-puberty)
Gu<br>PNAS<br>2015 (16)	8-22	780	motion regress. from mat.	no, but mat. norm. by μ	264	Pears. r	none	• variance of module size & similarity to adult modules incr.<br>• DMN: within + between-module strength increases<br>• hi.-order, subc./cerebell.: w/in + betw.-mod. strength decr.

Paper	Age (y)	# Sub.	Motion ⋆	GSR	Nodes	Edges	Thresh.	Results
Marek<br>PLoS CB<br>2015 (50)	10-26	192	wavelet<br>despike<br>+ scrub.	no	264	Pears.<br>r	binary<br>1-25%	• module organization stable<br>• cing.-operc./salience part. coeff. incr. (↦ inhibitory control)<br>• no distance-dependent change of strength
Kaufmann<br>Nat. Neurosci.<br>2017 (51)	8-22	797	AROMA<br>+ FIX	no	227<br>= 268<br>-41 drop.	Pears.<br>r	weighted	• resting-state, working memory task, emotion recognition task<br>• "fingerprinting" used to identify individual across scans<br>• identification accuracy ("distinctiveness") of indiv. FC increases
Kundu<br>J. Neurosci.<br>2018 (52)	8.3-46.2	51	ME-ICA	no	• voxels<br>• D-K	Pears.<br>r	weighted<br>r=0.3–0.9	• n° BOLD components decreases exponentially<br>• driven by dlPFC, parietal cortex, cerebellum<br>• BOLD dimensionality correlates to part. coeff.
Kolskar<br>eNeuro<br>2018 (53)	8-22	754	AROMA<br>+ FIX	no	voxels	ICA on<br>e-vector<br>cent.	N/A	• many sensorimotor & visual ICA components strengthen<br>• 2 fronto-parietal, 2 cingulo-opercular components strengthen<br>• 2 subcortical, 1 DMN ICA components weaken

Table S1. Summary of adolescent development of whole-brain resting-state functional networks. Only studies whose age-range presents overlap with the NSPN study of adolescent development (ages 14-24 years) were selected; studies on "lifespan" development whose age-range extends up to old age were not included. Studies exclusively focused on task fMRI, cognition or disease were not included. The list of included studies is not necessarily exhaustive; for further relevant discussion and references, see recent reviews on adolescent development including (54–57). ⋆ Methods for motion correction listed in the "Motion" column do not include linear regression of motion parameters and their derivatives (which was applied by most studies). ∗ The study by (21) (listed as Satterthw. PNAS 2014) is an arterial spin labeling (ASL) study.

Paper	Age (y)	# Sub.	Motion ⋆	GSR	Edges	hipp	amy	die	pal	put	acc	cau	thal
Qin<br>PNAS<br>2012 (58)	7-22	87	none	yes	mult.<br>regress^n		↑ para+limb.<br>ass^n<br>vmPFC						
Gabard-D.<br>NeuroIm.<br>2014 (59)	4-23	58	scrub.<br>+ FD reg.	yes	mult.<br>regress^n		↑ mPFC<br>↓ ins. / STS<br>parah. / PCC						
Greene<br>J. Neurosci.<br>2014 (60)	7-31	180	scrub.	no	partial<br>corr.					↓ somatomotor<br>face system<br>(BG: pal. + put.)			
Alarcon<br>NeuroIm.<br>2015 (61)	10-16	122	scrub.	yes	Pears. r		↓ par.-occip.<br>↓ mFC						
Fareri<br>NeuroIm.<br>2015 (62)	4.5-23	66	scrub.<br>+ FD reg.	yes	mult.<br>regress^n						ventral striatum<br>(put. + acc. + cau.)<br>↓ mPFC		
Sato<br>NeuroIm.<br>2015 (63)	7-15	447	scrub.	yes	Pears. r							↓ e-vec.<br>cent.	↓ e-vec.<br>cent.
van Duij.<br>NeuroIm.<br>2015 (64)	8-25	299	FD reg.	yes	dual<br>regress^n	↑ nucl. acc.					↑ dACC<br>hipp.	↑ dlPFC	↑ dlPFC
van Duij.<br>HBM<br>2019 (65)	8-29	273<br>661 scans<br>(longit.)	FIX	yes	Pears. r	↑ ACC<br>↑ nucl. acc.<br>↑ put. ↑ amy.	↑ put.			↑ front. med.<br>nucl. acc.	↑ ACC<br>front. med.	↑ ACC	

Table S2. Summary of adolescent development of subcortical resting-state functional networks. Only studies whose age-range presents substantial overlap with the NSPN study of adolescent development (ages 14-24 years) were selected; studies on "lifespan" development whose age-range extends up to old age were not included. Studies exclusively focused on task fMRI, cognition or disease were not included. The list of included studies is not necessarily exhaustive; for further relevant discussion and references, see recent reviews on adolescent development including (54–57). ⋆ Methods for motion correction listed in the "Motion" column do not include linear regression of motion parameters and their derivatives (which was applied by most studies).

Table S3. Maturation of subcortico-cortical FC. Frequencies of increases (↑) and decreases (↓) in subcortico-cortical FC as a function of age, for both the HCP and DK-sub cortical parcellations. These frequencies are listed as overall (all), and as numbers satisfying $P < 0.05$ - both without (P_{raw}) and with (P_{FDR}) correction of P-values for multiple comparisons. Subcortical regions are listed in decreasing order of average rate of change with age.

parcellation			amygdala	hippocampus	diencephalon	putamen	caudate	accumbens	pallidum	thalamus
HCP	↑	all	327	333	333	278	277	231	230	206
		P_{raw}	161	119	99	26	13	24	8	18
		P_{FDR}	69	5	0	0	0	0	0	0
	↓	all	19	13	13	68	69	115	116	140
		P_{raw}	0	0	0	0	2	2	2	0
		P_{FDR}	0	0	0	0	0	0	0	0
DK-sub	↑	all	278	287	289	230	238	203	193	163
		P_{raw}	132	106	71	14	10	11	2	13
		P_{FDR}	43	0	0	0	0	0	0	0
	↓	all	20	11	9	68	60	95	105	135
		P_{raw}	0	0	0	0	0	2	1	0
		P_{FDR}	0	0	0	0	0	0	0	0

Table S4. Relationships of maturational index with a range of cortical maps, in the main analysis and five sensitivity analyses. Map names on the left correspond to maps visualised in main text Fig. 4, and in supplementary Fig. S9. Columns contain the Spearman ρ, corresponding P-value as well as the P_{spin} P-value generated using the spatial permutation test. All P-values were adjusted for a total of 12 multiple comparisons (i.e., within columns) using the false discovery rate (FDR). For details regarding the sensitivity analyses, see *Sensitivity analyses* section.

analysis →	main results (520 scans)			DK-sub atlas (520 scans)			cross-sectional (298 scans)			single-scanner (396 scans)			low-motion (182 scans)			GSR (520 scans)			
map name ↓	ρ	P	P_{spin}	ρ	P	P_{spin}	ρ	P	P_{spin}	ρ	P	P_{spin}	ρ	P	P_{spin}	ρ	P	P_{spin}	ref.
Δ CT	0.16	0.0052	0.036	0.30	$1.3 \cdot 10^{-6}$	0.0093	0.14	0.013	0.049	0.15	0.0082	0.052	$\lvert< 0.01\rvert$	1	0.77	0.070	0.27	0.11	3
Δ $MT_{70\%}$	-0.079	0.15	0.33	-0.14	0.027	0.24	-0.097	0.094	0.30	-0.073	0.20	0.34	-0.043	0.52	0.48	-0.12	0.062	0.12	3
Δ SC_{CT}	0.096	0.098	0.20	0.031	0.61	0.38	0.070	0.22	0.27	0.068	0.22	0.26	< 0.01	1	0.68	0.030	0.59	0.4	4
glycolytic index	-0.56	$< 10^{-10}$	$< 10^{-4}$	-0.54	$< 10^{-10}$	$< 10^{-4}$	-0.51	$< 10^{-10}$	$< 10^{-4}$	-0.54	$< 10^{-10}$	$< 10^{-4}$	-0.32	$2.9 \cdot 10^{-8}$	0.011	-0.23	$2.3 \cdot 10^{-4}$	0.003	30
O_2 metabolic rate	-0.080	0.15	0.29	0.052	0.42	0.38	-0.023	0.68	0.42	-0.10	0.076	0.26	0.084	0.17	0.36	-0.098	0.11	0.12	30
glucose met. rate	-0.41	$< 10^{-10}$	0.0032	-0.33	$7.6 \cdot 10^{-8}$	0.019	-0.35	$8.0 \cdot 10^{-10}$	0.0098	-0.42	$< 10^{-10}$	0.0018	-0.16	0.0050	0.15	-0.21	$6.1 \cdot 10^{-4}$	0.016	30
cereb. blood vol.	0.29	$3.0 \cdot 10^{-7}$	0.033	0.24	$1.4 \cdot 10^{-4}$	0.076	0.26	$3.6 \cdot 10^{-6}$	0.036	-0.28	$1.1 \cdot 10^{-6}$	0.036	0.23	$6.0 \cdot 10^{-5}$	0.028	0.20	$9.7 \cdot 10^{-4}$	0.016	30
cereb. blood flow	-0.21	$1.8 \cdot 10^{-4}$	0.082	-0.16	0.011	0.14	-0.20	$6.4 \cdot 10^{-4}$	0.076	-0.24	$1.9 \cdot 10^{-5}$	0.052	-0.19	0.0012	0.061	-0.061	0.33	0.21	30
evolutionary exp.	-0.13	0.022	0.25	-0.26	$3.7 \cdot 10^{-5}$	0.059	-0.17	0.0026	0.17	-0.12	0.041	0.26	-0.31	$6.3 \cdot 10^{-8}$	0.0042	-0.034	0.59	0.38	33
postnatal exp.	-0.28	$8.7 \cdot 10^{-7}$	0.036	-0.26	$3.7 \cdot 10^{-5}$	0.059	-0.29	$5.0 \cdot 10^{-7}$	0.036	-0.25	$1.3 \cdot 10^{-5}$	0.052	-0.27	$4.0 \cdot 10^{-6}$	0.028	-0.13	0.04	0.11	33
areal scaling	-0.22	$9.0 \cdot 10^{-5}$	0.036	-0.18	0.0051	0.058	-0.21	$3.4 \cdot 10^{-4}$	0.036	-0.22	$1.1 \cdot 10^{-4}$	0.039	-0.12	0.052	0.15	-0.16	0.011	0.045	32
AG gene expr.	-0.34	$1.8 \cdot 10^{-5}$	0.0006	-0.23	0.010	0.019	-0.34	$1.7 \cdot 10^{-5}$	0.0015	-0.33	$3.6 \cdot 10^{-5}$	0.0012	-0.26	0.0015	0.010	-0.22	0.011	0.016	35

Neuroscience in Psychiatry Network (NSPN) Consortium author list

Principal investigators:

Edward Bullmore (CI from 01/01/2017)
Raymond Dolan
Ian Goodyer (CI until 01/01/2017)
Peter Fonagy
Peter Jones

NSPN (funded) staff:

Michael Moutoussis
Tobias Hauser
Sharon Neufeld
Rafael Romero-Garcia
Michelle St Clair
Petra Vértes
Kirstie Whitaker
Becky Inkster
Gita Prabhu
Cinly Ooi
Umar Toseeb
Barry Widmer
Junaid Bhatti
Laura Villis
Ayesha Alrumaithi
Sarah Birt
Aislinn Bowler
Kalia Cleridou
Hina Dadabhoy
Emma Davies
Ashlyn Firkins
Sian Granville
Elizabeth Harding
Alexandra Hopkins
Daniel Isaacs
Janchai King
Danae Kokorikou
Christina Maurice
Cleo McIntosh
Jessica Memarzia
Harriet Mills
Ciara O'Donnell
Sara Pantaleone
Jenny Scott

Affiliated scientists:

Pasco Fearon
John Suckling
Anne-Laura van Harmelen
Rogier Kievit

References

1. Barth M, Reichenbach JR, Venkatesan R, Moser E, Haacke EM (1999) High-resolution, multiple gradient-echo functional MRI at 1.5 T. *Magn. Reson. Imaging* 17(3):321–329.
2. Fischl B, Sereno M, Dale A (1999) Cortical surface-based analysis. II: Inflation, flattening, and a surface-based coordinate system. *Neuroimage* 9(2):195–207.
3. Whitaker KJ, et al. (2016) Adolescence is associated with transcriptionally patterned consolidation of the hubs of the human brain connectome. *Proc. Natl. Acad. Sci. U. S. A.* 113(32):9105–9110.
4. Váša F, et al. (2018) Adolescent Tuning of Association Cortex in Human Structural Brain Networks. *Cereb. Cortex* 28(1):281—-294.
5. Saad ZS, et al. (2009) A new method for improving functional-to-structural MRI alignment using local Pearson correlation. *Neuroimage* 44(3):839–848.
6. Kundu P, Inati SJ, Evans JW, Luh WM, Bandettini Pa (2012) Differentiating BOLD and non-BOLD signals in fMRI time series using multi-echo EPI. *Neuroimage* 60(3):1759–70.
7. Kundu P, et al. (2013) Integrated strategy for improving functional connectivity mapping using multiecho fMRI. *Proc. Natl. Acad. Sci. U. S. A.* 110(40):16187–92.
8. Posse S, et al. (1999) Enhancement of BOLD-contrast sensitivity by single-shot multi-echo functional MR imaging. *Magn. Reson. Med.* 42(1):87–97.
9. Power JD, Barnes KA, Snyder AZ, Schlaggar BL, Petersen SE (2012) Spurious but systematic correlations in functional connectivity MRI networks arise from subject motion. *Neuroimage* 59(3):2142–2154.
10. Patel AX, et al. (2014) A wavelet method for modeling and despiking motion artifacts from resting-state fMRI time series. *Neuroimage* 95:287–304.
11. Glasser MF, et al. (2016) A multi-modal parcellation of human cerebral cortex. *Nature* pp. 1–11.
12. Desikan RS, et al. (2006) An automated labeling system for subdividing the human cerebral cortex on MRI scans into gyral based regions of interest. *Neuroimage* 31(3):968–980.
13. Romero-Garcia R, Atienza M, Clemmensen LH, Cantero JL (2012) Effects of network resolution on topological properties of human neocortex. *Neuroimage* 59(4):3522–3532.
14. Filipek PA, Richelme C, Kennedy DN, Caviness VS (1994) The Young Adult Human Brain: An MRI-based Morphometric Analysis. *Cereb. Cortex* 4(4):344–360.
15. Satterthwaite TD, et al. (2012) Impact of in-scanner head motion on multiple measures of functional connectivity: Relevance for studies of neurodevelopment in youth. *Neuroimage* 60(1):623–632.
16. Gu S, et al. (2015) Emergence of system roles in normative neurodevelopment. *Proc. Natl. Acad. Sci. U. S. A.* 112(44):13681–13686.
17. Fjell AM, et al. (2010) When does brain aging accelerate? Dangers of quadratic fits in cross-sectional studies. *Neuroimage* 50(4):1376–83.
18. Reiss PT, et al. (2014) Massively parallel nonparametric regression, with an application to developmental brain mapping. *J. Comput. Graph. Stat.* 23(1):232–248.
19. von Economo C, Koskinas GN (1925) *Die Cytoarchitektonik der Hirnrinde des Erwachsenen Menschen: Textband und Atlas mit 112 Mikrophotographischen Tafeln.* (Springer, Vienna).
20. Yeo BTT, et al. (2011) The organization of the human cerebral cortex estimated by intrinsic functional connectivity. *J. Neurophysiol.* 106(3).
21. Satterthwaite TD, et al. (2014) Impact of puberty on the evolution of cerebral perfusion during adolescence. *Proc. Natl. Acad. Sci. U. S. A.* 111(23):8643–8.
22. Alexander-Bloch A, Raznahan A, Bullmore E, Giedd J (2013) The convergence of maturational change and structural covariance in human cortical networks. *J. Neurosci.* 33(7):2889–99.
23. Vandekar SN, et al. (2015) Topologically Dissociable Patterns of Development of the Human Cerebral Cortex. *J. Neurosci.* 35(2):599–609.
24. Lefèvre J, et al. (2018) SPANOL (SPectral ANalysis of Lobes): A Spectral Clustering Framework for Individual and Group Parcellation of Cortical Surfaces in Lobes. *Front. Neurosci.* 12:1–14.
25. Blaser R, Fryzlewicz P (2016) Random rotation ensembles. *J. Mach. Learn. Res.* 17:1–26.
26. Vértes P, et al. (2016) Gene transcription profiles associated with inter-modular hubs and connection distance in human functional magnetic resonance imaging networks. *Philos. Trans. R. Soc. B Biol. Sci.* 371(1705).
27. Ad-Dab'bagh Y, et al. (2006) The CIVET Image-Processing Environment: A Fully Automated Comprehensive Pipeline for Anatomical Neuroimaging Research in *Proc. 12th Annu. Meet. Organ. Hum. Brain Mapp.*
28. Yarkoni T, Poldrack RA, Nichols TE, Van Essen DC, Wager TD (2011) Large-scale automated synthesis of human functional neuroimaging data. *Nat. Methods* 8(8):665–670.
29. Cox RW (1996) AFNI: software for analysis and visualization of functional magnetic resonance neuroimages. *Comput. Biomed. Res.* 29(3):162–73.
30. Vaishnavi SN, et al. (2010) Regional aerobic glycolysis in the human brain. *Proc. Natl. Acad. Sci. U. S. A.* 107(41):17757–17762.
31. Glasser MF, Goyal MS, Preuss TM, Raichle ME, Van Essen DC (2014) Trends and properties of human cerebral cortex: Correlations with cortical myelin content. *Neuroimage* 93:165–175.

32. Reardon PK, et al. (2018) Normative brain size variation and brain shape diversity in humans. *Science* 360(6394):1222–1227.
33. Hill J, et al. (2010) Similar patterns of cortical expansion during human development and evolution. *Proc. Natl. Acad. Sci. U. S. A.* 107(29):13135–40.
34. Goyal MS, Hawrylycz M, Miller JA, Snyder AZ, Raichle ME (2014) Aerobic glycolysis in the human brain is associated with development and neotenous gene expression. *Cell Matab* 19(2):49–57.
35. Hawrylycz MJ, Lein ES, Guillozet-Bongaarts AL, Shen EH, Ng Lea (2012) An anatomically comprehensive atlas of the adult human brain transcriptome. *Nature* 489(7416):391–399.
36. Arnatkeviciute A, Fulcher BD, Fornito A (2019) A practical guide to linking brain-wide gene expression and neuroimaging data. *Neuroimage* 189:353–367.
37. Romero-Garcia R, et al. (2018) Structural covariance networks are coupled to expression of genes enriched in supragranular layers of the human cortex. *Neuroimage* 171:256–267.
38. Power JD, et al. (2018) Ridding fMRI data of motion-related influences: Removal of signals with distinct spatial and physical bases in multiecho data. *Proc. Natl. Acad. Sci. U. S. A.* 115(9):E2105–E2114.
39. Murphy K, Fox MD (2017) Towards a consensus regarding global signal regression for resting state functional connectivity MRI. *Neuroimage* 154:169–173.
40. Fair D, et al. (2007) Development of distinct control networks through segregation and integration. *Proc. Natl. Acad. Sci. U. S. A.* 104(33):13507–12.
41. Fair D, et al. (2009) Functional brain networks develop from a "local to distributed" organization. *PLoS Comput. Biol.* 5(5):e1000381.
42. Supekar K, Musen M, Menon V (2009) Development of large-scale functional brain networks in children. *PLoS Biol.* 7(7):e1000157.
43. Stevens MC, Pearlson GD, Calhoun VD (2009) Changes in the interaction of resting-state neural networks from adolescence to adulthood. *Hum. Brain Mapp.* 30(8):2356–2366.
44. Dosenbach NUF, et al. (2010) Prediction of individual brain maturity using fMRI. *Science* 329(5997):1358–1361.
45. Anderson JS, Ferguson MA, Lopez-Larson M, Yurgelun-Todd D (2011) Connectivity Gradients Between the Default Mode and Attention Control Networks. *Brain Connect.* 1(2):147–157.
46. Satterthwaite TD, et al. (2013) Heterogeneous impact of motion on fundamental patterns of developmental changes in functional connectivity during youth. *Neuroimage* 83:45–57.
47. Hwang K, Hallquist MN, Luna B (2013) The development of hub architecture in the human functional brain network. *Cereb. Cortex* 23(10):2380–2393.
48. Wu K, et al. (2013) Topological organization of functional brain networks in healthy children: differences in relation to age, sex, and intelligence. *PLoS One* 8(2):e55347.
49. Grayson DS, et al. (2014) Structural and functional rich club organization of the brain in children and adults. *PLoS One* 9(2):1–13.
50. Marek S, Hwang K, Foran W, Hallquist MN, Luna B (2015) The Contribution of Network Organization and Integration to the Development of Cognitive Control. *PLoS Biol.* 13(12):1–25.
51. Kaufmann T, et al. (2017) Delayed stabilization and individualization in connectome development are related to psychiatric disorders. *Nat. Neurosci.* 20(May 2016):513—515.
52. Kundu P, et al. (2018) The Integration of Functional Brain Activity from Adolescence to Adulthood. *J. Neurosci.* 38(14):3559–3570.
53. Kolskår KK, et al. (2018) Key brain network nodes show differential cognitive relevance and developmental trajectories during childhood and adolescence. *eNeuro* 5(4).
54. Vertes PE, Bullmore ET (2015) Annual research review: Growth connectomics - the organization and reorganization of brain networks during normal and abnormal development. *Journal of Child Psychology and Psychiatry* 56(3):299–320.
55. Stevens MC (2016) The contributions of resting state and task-based functional connectivity studies to our understanding of adolescent brain network maturation. *Neurosci. Biobehav. Rev.* 70:13–32.
56. Morgan SE, White SR, Bullmore ET, Vértes PE (2018) A Network Neuroscience Approach to Typical and Atypical Brain Development. *Biol. Psychiatry Cogn. Neurosci. Neuroimaging* 3(9):754–766.
57. Foulkes L, Blakemore SJ (2018) Studying individual differences in human adolescent brain development. *Nat. Neurosci.* 21:315—-323.
58. Qin S, Young CB, Supekar K, Uddin LQ, Menon V (2012) Immature integration and segregation of emotion-related brain circuitry in young children. *Proc. Natl. Acad. Sci. U. S. A.* 109(20):7941–7946.
59. Gabard-Durnam LJ, et al. (2014) The development of human amygdala functional connectivity at rest from 4 to 23 years: A cross-sectional study. *Neuroimage* 95:193–207.
60. Greene DJ, et al. (2014) Developmental Changes in the Organization of Functional Connections between the Basal Ganglia and Cerebral Cortex. *J. Neurosci.* 34(17):5842–5854.
61. Alarcón G, Cservenka A, Rudolph MD, Fair DA, Nagel BJ (2015) Developmental sex differences in resting state functional connectivity of amygdala sub-regions. *Neuroimage* 115:235–244.
62. Fareri DS, et al. (2015) Normative development of ventral striatal resting state connectivity in humans. *Neuroimage* 118:422–437.
63. Sato JR, et al. (2015) Decreased centrality of subcortical regions during the transition to adolescence: A functional

connectivity study. *Neuroimage* 104C:44–51.

64. Van Duijvenvoorde ACK, Achterberg M, Braams BR, Peters S, Crone EA (2015) Testing a dual-systems model of adolescent brain development using resting-state connectivity analyses. *Neuroimage* 124:409–420.
65. van Duijvenvoorde AC, Westhoff B, de Vos F, Wierenga LM, Crone EA (2019) A three-wave longitudinal study of subcortical–cortical resting-state connectivity in adolescence: Testing age- and puberty-related changes. *Hum. Brain Mapp.* (April):1–15.
